# Betelvine (*Piper betle* L.): A comprehensive insight into its ethnopharmacology, phytochemistry, and pharmacological, biomedical and therapeutic attributes

**DOI:** 10.1111/jcmm.17323

**Published:** 2022-05-02

**Authors:** Protha Biswas, Uttpal Anand, Suchismita Chatterjee Saha, Nishi Kant, Tulika Mishra, Harison Masih, Ananya Bar, Devendra Kumar Pandey, Niraj Kumar Jha, Madhumita Majumder, Neela Das, Vijaykumar Shivaji Gadekar, Mahipal S. Shekhawat, Manoj Kumar, Radha, Jarosław Proćków, José M. Pérez de la Lastra, Abhijit Dey

**Affiliations:** ^1^ Department of Life Sciences Presidency University Kolkata West Bengal India; ^2^ 26732 Department of Life Sciences Ben‐Gurion University of the Negev Beer‐Sheva Israel; ^3^ Department of Zoology Nabadwip Vidyasagar College (Affiliated to the University of Kalyani) Nabadwip West Bengal India; ^4^ Department of Biotechnology School of Health and Allied Science ARKA Jain University Jamshedpur Jharkhand India; ^5^ Department of Botany Deen Dayal Upadhyay Gorakhpur University Gorakhpur Uttar Pradesh India; ^6^ Department of Industrial Microbiology Jacob Institute of Biotechnology and Bioengineering Sam Higginbottom University of Agriculture, Technology and Sciences Prayagraj Uttar Pradesh India; ^7^ Department of Zoology Wilson College (Affiliated to University of Mumbai) Mumbai Maharashtra India; ^8^ 126208 Department of Biotechnology Lovely Professional University Phagwara Punjab India; ^9^ 193167 Department of Biotechnology School of Engineering & Technology Sharda University Greater Noida Uttar Pradesh India; ^10^ Department of Botany Raidighi College (Affiliated to University of Calcutta) Raidighi West Bengal India; ^11^ Department of Botany Rishi Bankim Chandra College (Affiliated to the West Bengal State University) Naihati West Bengal India; ^12^ Zoology Department Sangola College (Affiliated to Punyashlok Ahilyadevi Holkar Solapur University) Solapur Maharashtra India; ^13^ Plant Biotechnology Unit Kanchi Mamunivar Government Institute for Postgraduate Studies and Research Puducherry India; ^14^ Chemical and Biochemical Processing Division ICAR ‐ Central Institute for Research on Cotton Technology Mumbai Maharashtra India; ^15^ School of Biological and Environmental Sciences Shoolini University of Biotechnology and Management Sciences Solan Himachal Pradesh India; ^16^ 56641 Department of Plant Biology Institute of Environmental Biology Wrocław University of Environmental and Life Sciences Wrocław Poland; ^17^ Instituto de Productos Naturales y Agrobiología (IPNA) Consejo Superior de Investigaciones científicas (CSIS) Santa Cruz de Tenerife Spain

**Keywords:** Betelvine (*Piper betle* L.), ethnobotany, hydroxychavicol, nanoparticles, pharmacology, phytochemicals

## Abstract

*Piper betle* L. (synonym: *Piper betel* Blanco), or betel vine, an economically and medicinally important cash crop, belongs to the family Piperaceae, often known as the green gold. The plant can be found all over the world and is cultivatedprimarily in South East Asian countries for its beautiful glossy heart‐shaped leaves, which are chewed or consumed as betelquidand widely used in Chinese and Indian folk medicine, as carminative, stimulant,astringent, against parasitic worms, conjunctivitis, rheumatism, wound, etc., andis also used for religious purposes. Hydroxychavicol is the most important bioactive compound among the wide range of phytoconstituents found in essential oil and extracts. The pharmacological attributes of *P*.* betle* are antiproliferation, anticancer, neuropharmacological, analgesic, antioxidant, antiulcerogenic, hepatoprotective, antifertility, antibacterial, antifungal and many more. Immense attention has been paid to nanoformulations and their applications. The application of *P*.* betle* did not show cytotoxicity in preclinical experiments, suggesting that it could serve as a promising therapeutic candidate for different diseases. The present review comprehensively summarizes the botanical description, geographical distribution, economic value and cultivation, ethnobotanical uses, preclinical pharmacological properties with insights of toxicological, clinical efficacy, and safety of *P*.* betle*. The findings suggest that *P*.* betle* represents an orally active and safe natural agent that exhibits great therapeutic potential for managing various human medical conditions. However, further research is needed to elucidate its underlying molecular mechanisms of action, clinical aspects, structure–activity relationships, bioavailability and synergistic interactions with other drugs.

## 1 INTRODUCTION

*Piper betle* L. (synonym: *Piper betel* Blanco) (Piperaceae) is a widely known perennial creeping plant belonging to the genus Piperaceae and originates from central and eastern Peninsular Malaysia and is distributed to East Africa and tropical countries of Asia.^1^ It is a commercial cash crop cultivated mainly in India, Bangladesh, Sri Lanka Thailand, Taiwan, Malaysia and few other Southeast Asian countries.^2^, ^3^ The betelvine is called the ‘green gold of India’ because almost 20 million people depend on this plant to derive their source of income from the production, transportation, handling, processing and preparation of betel leaves.^4^, ^5^ The betel vine is usually an asexually propagated plant that has various cultivars and bears both male and female plants. About a hundred varieties of betel plants are found across the world, among them 40 varieties are found only in India and of which 30 are recorded from West Bengal and Bangladesh.^6^ The most common varieties of betel are Magadhi, Salem, Mysore, Bangla, Kauri, Venmony, Meetha, Kapoori, Sanchi, Banarasi, Desavari, Kasi, Ghanagete and Bagerhati, which are mainly based upon their colour, aroma, taste and size.^1^ *P*.* betle* is known by various names in different countries around used globe, though ‘Paan’ is the most used in India, Pakistan, Nepal and Bangladesh.^7^ The betel leaf and areca nuts play a central role in Hindu culture as they are used in a variety of social, cultural and religious ceremonies.^1^ Betel quid is a common practice in many countries because it acts as a natural tonic and mouth refresher to prevent oral malodour. The International Agency for Research on Cancer surveyed and estimated that there are 200–600 million users present globally (Refs ^251^, ^271^; IARC).

The use of *P*.* betle* is found in many traditional medicinal systems, such as the Indian Ayurvedic medicinal system, traditional Chinese medicine, and also in the folklore medicinal system of the West Indies and Latin America. In the Ayurvedic medicine system, *P*.* betle* plants are used as preparation varieties for the treatment of many diseases, known as Lokantha Rasa, Puspadhava Rasa, Laghu‐sutaseknara Rasa, Lanha, Brhat sarwajwarahara and Brhat visamaj warantaka Rasa. The juice prepared from the betle leaf is generally used as an adjuvant in many herbal combinations with different other medicinal plants for better results in Ayurveda.^8^ Traditionally, the plant is used to cure many ailments such as cold, bronchial asthma, cough, stomachalgia and rheumatism, and it is used for the treatment of other diseases such as boils, bad breath, constipation, conjunctivitis, gum swelling, abscesses, injuries and cuts, which are communicable or noncommunicable.^9^ The use of this plant is also found in other purposes, such as in fish poisoning, fish bait, insecticides, ornaments, oils, perfumes and hallucinogens.^10^

Pharmacological properties of medicinal plants are primarily attributed to a variety of bioactive phytochemicals with biomedical and pharmaceutical significance.^11^, ^12^, ^13^, ^14^, ^15^, ^16^, ^17^, ^18^ Plants are known to house a number of different classes of phytoconstituents^19^, ^20^, ^21^ such as alkaloids, glycosides, tannins, phenolic compounds, flavonoids, terpenes and oligosaccharides.^22^, ^23^, ^24^ Such phytochemicals have also been reported against an array of human ailments.^20^, ^21^, ^25^ The strong pungent aroma comes from the leaves of betel because the essential oil contains a good quantity of terpenes and phenols. ^26^, ^27^, ^28^ The essential oil from betel leaf is to some extent a greasy, slippery and viscous liquid at room temperature. A wide diversity of bioactive compounds is present in the leaves of betel, this difference is based on the environment, soil types, the location of growing and types of landraces.^7^ The wide range of phytochemicals present in the betel plants was identified as chavicol, chavibetol, hydroxychavicol, eugenol, estragole, methyl eugenol, hydroxycatechol, α‐pinene, caryophyllene, β‐pinene, 1,8‐cineol and others.^29^ In a recent report, combining herbs (*P*.* betle* leaves) and herbo‐minerals (Swarna bhasma) in a dosage‐dependent manner was used to treat Corona patients by enhancing their prophylactic and therapeutic effects.^30^

Various extraction methods are used for the extraction of volatile oils from the leaves of betel, including hydrodistillation, steam distillation, solvent extraction and supercritical fluid extraction that were characterized by GC‐MS, NMR.^1^ Huge numbers of studies have revealed the efficacy of the bioactive compounds present in essential oil as antioxidants to prevent cancer, inflammation, neurodegenerative disorders, and also as antimutagenic, antifertility, antilipidaemic, antiglycaemic, cardioprotective, etc.^31^ ^,^ ^32^ The essential oil of betel leaves can also combat bacterial, protozoan and fungal infections and insect attacks.

This review comprehensively summarizes the botanical description, economic status, pharmacological properties, nanoformulations and their applications taking into account the safety and toxicity. In addition, the underlying molecular basis of the action of plant extracts or phytochemicals are also discussed. Considering the immense potential of this underexploited medicinal plant, the present review comprehensively describes the present state of the art research on this plant with an interdisciplinary approach that includes the pharmacology, nanotechnology, preclinical and clinical studies and also potential toxicological considerations of using *P*.* betle* preparations. However, more studies are needed to enumerate the structure–activity relationships behind the pharmacological activity of plant constituents. Detailed clinicalstudies are also needed, and the pharmacokinetic properties and druggability ofsuch preparations need to be elucidated.

## 2 TAXONOMY

Taxonomical classification

Kingdom: Plantae

Division: Magnoliophyta

Class: Magnoliopsida

Order: Piperales

Family: Piperaceae

Genus: *Piper*

Species: *Piper betle* L.^33^

## 3 BOTANICAL DESCRIPTION

**FIGURE 1:**
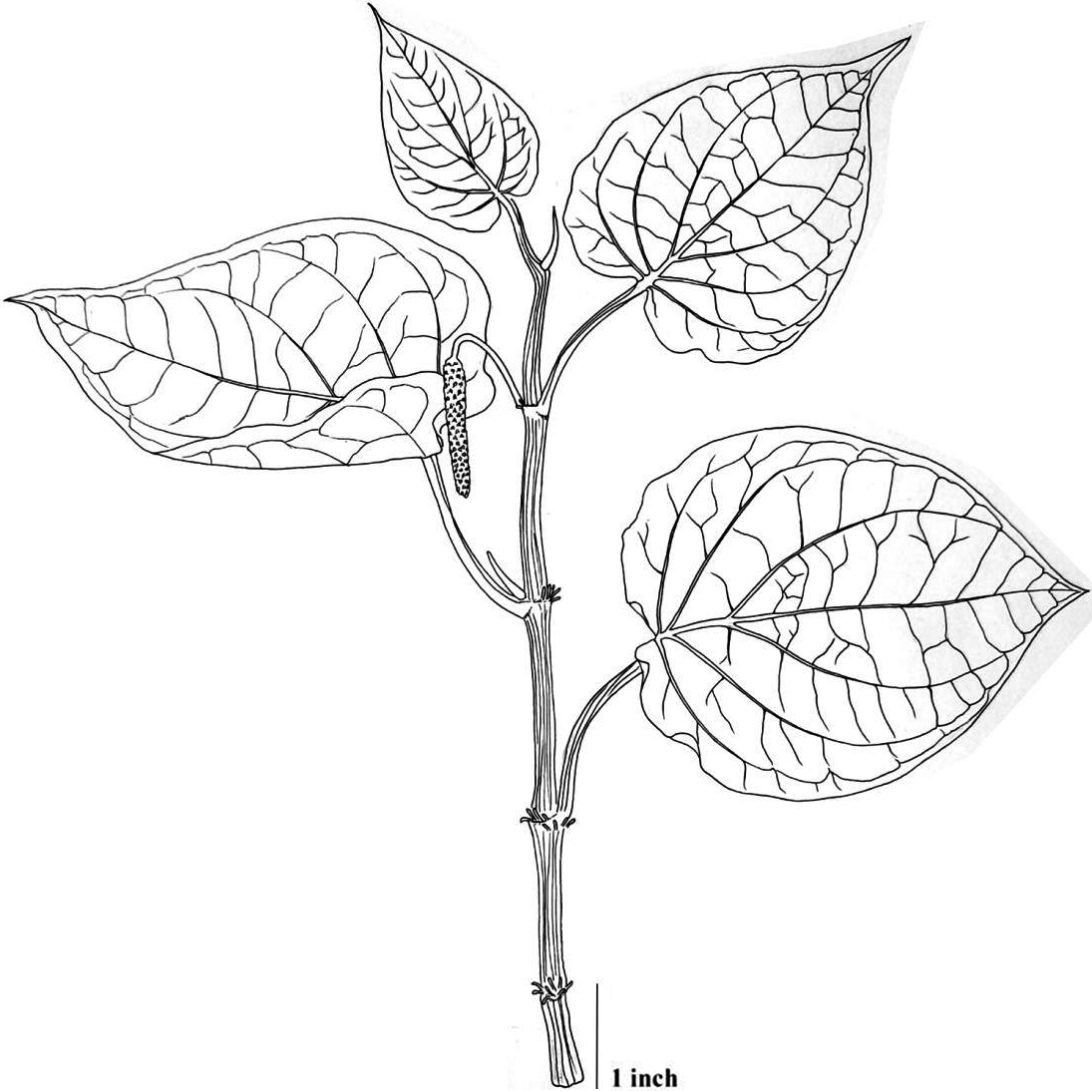
*Piper betle* L.: Habit sketch

The plant (Figure 1) is a dioecious root climber, and the shoots reach any height from 3 to 10 m according to available facilities for climbing. The plant bears lateral branches along its entire length that grow a couple of feet from the ground. The stems are swollen and articulate, with dichotomous branching and rooting at the nodes. The stems are stout, almost terete, slightly flattened; when young, they are light green and marked by short, raised, whitish streaks and with pinkish stripes along the node. The internodes generally attain a length of about 12 cm. and a diameter of 1.2 cm. Leaves are characterized as a simple blade, alternate, spiral and ex‐stipulate; petioles are 2–5 mm long, pubescent and channelled. Leaf blades are glabrous, coriaceous, fleshy, greenish to yellowish, shining, broadly ovate, width 7–8.5 cm, length 9–11 cm; base cordate; apex acuminate; margin is entire, narrowly recurved; venation reticulate, 7–9 veins in two or three pairs coming from the midrib, one pair elevating from base. The inflorescence is an axillary spike up to 5.5 cm long. The male inflorescence forms a cylindrical pendulous catkin of 10 cm in length and 2 cm in diameter. Female spikes are also cylindrical, pendulous; length 2.5–4 cm and diameter 0.5 cm. Individual flowers are very minute and unisexual, reduced, consisting of a couple of stamens and stigmas inserted into the axil of each bract. The bracts are orbicular, peltate, arranged in a thickly crowded spiral series. The mature inflorescence is strongly aromatic. Fruiting spikes are 3–5 cm in length, orange and drupping, entrenched on the rachis of the mature inflorescence.^34^ ^,^ ^35^

## 4 VERNACULAR NAME

Vernacular names in Indian languages

Sanskrit: Tambool, Mukhbhushan, Nagavalli, Varnalata, Nagavallari

Hindi, Bengali, Urdu: Paan

Telugu: Nagballi, Tamalapaku

Tamil: Vetrilai

Gujarati: Nagarbael

Marathi: Vidyache pan

Malayalam: Vettilakkoti, Vettila

Kannada: Veeleya, Veeleyada yele, Vilya, Villayadel

Konkani: Phodi paan

Other Asian languages

English: Betle, Betle pepper, Betle‐vine

Vietnamese: Tråu

Khmer: Maluu

Mon: Plu

Thai: Plue, Pelu

Persian: Burg‐e‐Tanbol

Chamorro: Papulu

Javanese: Suruh, Sirih, Bodeh

Arabic: Tanbol

Sakai: Jerak

Semang: Seresa, Be, Cabe

Sinhalese: Bulath

Jakun: Kerekap, Kenayek

Malay: Daun sirih, Sirih hudang, Sirih Carang, Sirih melayu

Kapampangan: Bulung samat^36^ ^,^ ^37^

## 5 DISTRIBUTION AND CULTIVATION

**FIGURE 2:**
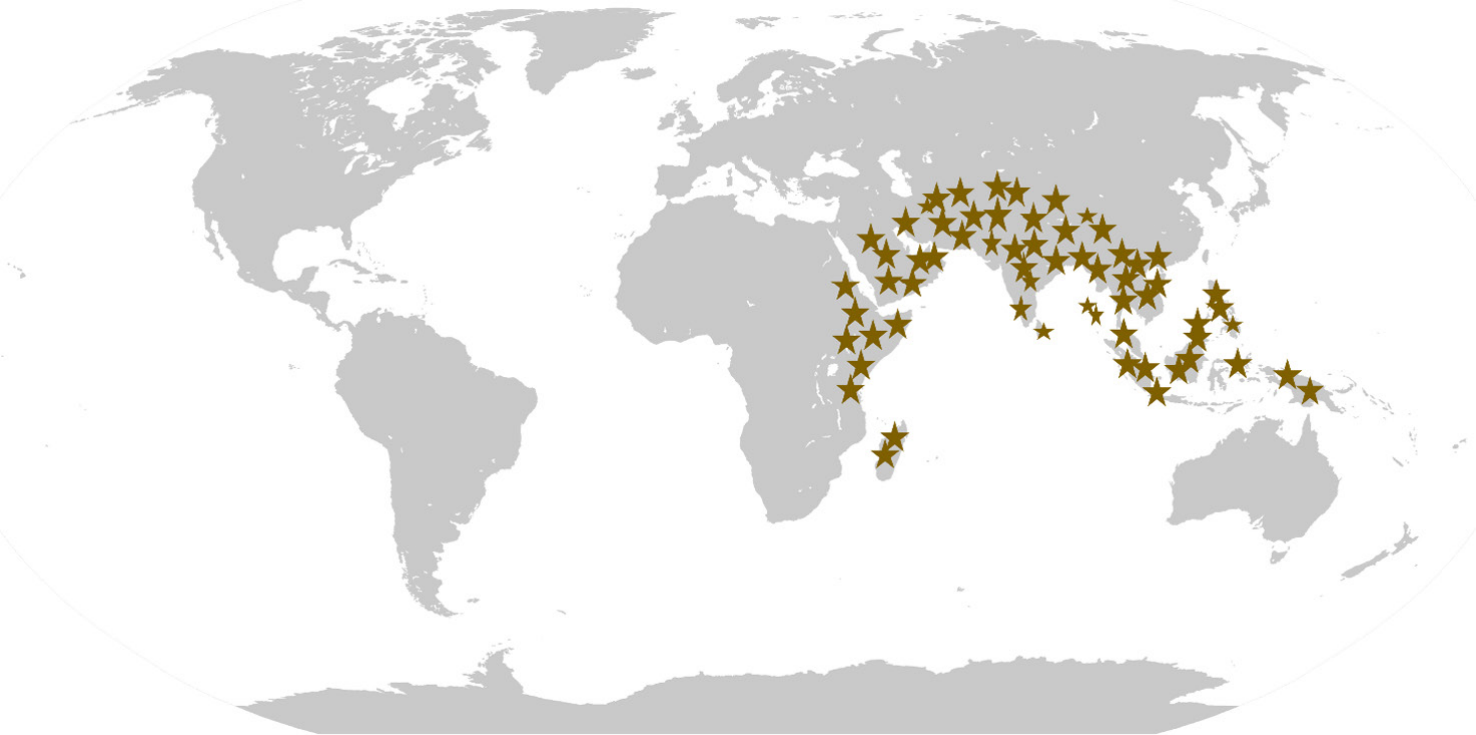
Geographical distribution of *Piper betle* L. throughout the world

The betel vine is believed to have originated in Malaysia.^38^ The plant is widely grown in forests that are generally damp and also in hot and moist climatic conditions of India and many other countries in South and South East Asia, viz. China and Vietnam. *Piper betle* is believed to have first emerged in tropical Asia and then spread to East Africa and Madagascar. Betel is widely grown in India, Sri Lanka, Bangladesh, Indonesia, Nepal, Pakistan, Vietnam, Thailand, Laos, Kampuchea, Philippine Islands, Burma, Malaysia, Taiwan, Malay Peninsula and many countries in Southeast Asia and is known to have a long history, mentioning the presence of the betel plant over 2000 years. ^39^Figure 2 presents the worldwide distribution of the species. In India, the plant is found in Bengal, Bihar, Orissa, Andhra Pradesh, Karnataka, Uttar Pradesh and Tamilnadu.^40^, ^41^, ^42^ About a hundred betel plant varieties are found all over the world,among them, 40 are grown in India, along with 30 are grown in Bangladesh and West Bengal.^43^ Various types of *Piper betle* are found across the world, for instance, Magadhi, Kauri, Meetha, Salem, Venmony, Bangla, Banarasi, Kapoori, Kasi, Sanchi, Mysore, Desavari, Ghanagete and Bagerhati according to their size, colour and aroma.^44^

*Piper betle* is generally propagated asexually by cutting stems rather than germinating seeds.^45^ It needs a compatible tree or long support for its creeping habit. Betel vine cultivation is a very typical type of farming. For betel cultivation, the best choices are highlands and especially fertile sandy or sandy clay or sandy loam soil with a well drainage system and a pH range of 5.6–8.2, thus, saline and alkali soils where water logging are a problem is not suitable; about 2250–4750 mm rainfall, relative humidity 40–80% and temperature range 15–40°C are considered suitable. In Bangladesh, farmers prepare a special garden called ‘barouj’ which is fenced with bamboo sticks and coconut leaves and on top of the fench is covered with paddy leaves to grow betel. The farming land is well dug into furrows of approximately 10–15 m long, 75 cm wide and 75 cm deep. The furrows are thoroughly manured with cow dung, rotten farmyard manure, oil cakes, leaves and wood ash. After proper dressing, the cuttings are planted at the beginning of the monsoon, in the months of May to June. Then, the plants are parallelly arranged in rows with a distance of two feet between each plant and are bound with a string around upright sticks of split bamboo or short plants for support. Proper shade and frequent irrigation are necessary in areas where rainfall is lower about 1500–1700 mm; regular watering is required in summer and watering every 3–4 days is sufficient in winter, and a proper drainage system is mandatory at the time of rainy season for the successful cultivation of this crop. After 1 year of planting, the leaves of the plant turn out to be ready for plucking, and the production of betel leaf from the barouj lasts for more than a few years from the time of planting.^41^ ^,^ ^43^ ^,^ ^46^

## 6 ECONOMIC STATUS

In the Indian climate, the female plants of *Piper betle* rarely produce flowers or fruit. Betel vines are cultivated and harvested mainly for their heart‐shaped green leaves.^41^ This crop has a vast economic potentiality which can be effectively recognized by the piece of evidence that more or less 15–20 million of people in India have the habit of using betel leaves regularly^47^ not only that, there are more than 2 billion people from many other countries who are recognized as regular users of betel leaves from all over the world.^48^ Most importantly, the economic status of betel leaves is dependent on the physical character of the end products in the worldwide market. The betel leaf and products produced in different forms such as powder, capsules, liquid and various types of value‐added products are available on a broad spectrum in the market as beverages, in oral care, pharmaceutical products and cosmetics.^41^ The annual turnover national income is Rs 7000–10,000 million, and from this, the state, West Bengal, gains an income of 800–1000 million rupees per year. The leaves were exported to various countries around the world where the plant is not grown naturally or the local supply could not meet the requirements. Betel leaves are generally exported to Hong Kong, Pakistan, Italy, Bahrain, Canada, Great Britain, Kuwait, Saudi Arab, Nepal and several other countries in Europe.^47^ ^,^ ^49^

## 7 TRADITIONAL AND ETHNO‐MEDICINAL USE

Traditional medicine has played a crucial role in the health care of the rural and urban people.^246^, ^247^, ^248^ Ethno‐medico‐botanicals have been used across almost all the cultures worldwide against an array of human medical conditions.^50^, ^51^, ^52^, ^53^ The use of betel leaf alone and with a combination of other plants or medicines for better therapeutic effects is mentioned in the Ayurvedic literature, which was almost 1400 BC ago. ^54^Atharved, the ancient Vedic literature, mentioned the usefulness of the betel plant against numerous diseases at about 3000–2500 BC before.^55^ Saptasira, the Vedic name of the leaves of betel, is mentioned in the Kamasutra of Vatsyayan as having aphrodisiac properties.^44^ In the ayurvedic and Unani system of medicine, the betel plant is used as an anthelmintic, appetite stimulant, vermifuge, astringent, diarrhoea, aphrodisiac, breath freshener, carminative, cardiac tonic, dentifrice, in the prevention of diuretic emmenagogues, induction and increase of menstrual flow, laxative, strengthen gums, nerve tonic and also in the treatment of urinary disorders. Betel leaves are mostly chewed by about 200 million people on a regular basis throughout the south Asia and western part of the Pacific basin in a special shape of packets known as ‘Betel quid’, which is prepared from *Piper betle* leaves brushed with burnt lime and contain few pieces of areca nut, flavours, often cardamom or cloves, are added with or without tobacco according to choice.^56^ Chaveerach et al. stated that the betel leaf is a most important material in Thai ceremonies. Elderly people chew betel leaves to prepare quid. In weddings, the family members of the bridegroom place money along with the *betle* leaves in a bowl, which together is known as khun maak. The ethnic group Kui, from the southern division of North East Thailand, uses betel leaf (locally, raam phi taan) in the ‘Spirit dancing’ ceremony to chase away evil spirits or fend off bad luck from the patients from the family or the village. They use betel leaves as stimulant, exhilarant, antiseptic and antioxidant, to treat kidney inflammation and thirst resulting from diabetes, strength to stomach, as expectorant for asthma, coughs and bronchitis, and antiflatulent element.^34^ Decoction of *P*.* betle* leaves used to prevent body odour and treat diarrhoea, sore throat, skin allergies and fluor albus, leaves are cooked and added to vegetable soup.^57^ In Southeast Asia, Betel chewing with its associated discoloration of the teeth is the ascriptions of the teeth blackening practice related to sexual maturation and becoming a full member of society in Masticans.^58^ In the Laleng community, people use betel leaf to chew and at the sociocultural festival. They oil the leaf with mustard oil and place it on the naval area to relieve liver pain.^59^ The Rabha community of Mataikhar forest, Assam, the Torajanese, the Bugis community and Lakshadweep people also use betel leaf for chewing and in religious festivals.^60^, ^61^, ^62^, ^63^ People in Parsa district, Nepal, chew betel leaf or mix leaf juice with hot water, honey or milk mild stimulant, cure worm, remedy for bad breath and provides mouth refreshment, improve digestion, strengthen teeth and gums, palate cleaner, treatment of nervous pains and exhaustion, ease of urination, analgesic, reduce cough and cold.^64^ The ethno‐medicinal uses of *P*.* betle* in the area and community are listed in Table 1.

**TABLE 1:** Ethnomedicinal uses of *P*.* betle*

Local name	Community/tribe and region	Part used and preparation	Medicinal property/used against	Reference
Paan	Dibru‐Saikhowa Biosphere Reserve of Northeast India	leaf infusion	abdominal pain	^267^
Daing	Kadazandusun communities around Crocker range	leaf tea taken orally, paste applied topically	cough, scabies, boils, nosebleed	^239^
–	Thailand	leaves	stimulant, exhilarant, antiseptic and antioxidant, kidney inflammation and thirst resulting from diabetes, strength to stomach, expectorant effect for coughs, asthma and bronchitis, antiflatulent material	^34^
paan	Assam	crushed leaf juice	pediculosis	^269^
Tamalapaku	Andhra Pradesh, India	leaves	asthma	^271^
Paan	Garo tribal community, Netrakona district, Bangladesh	paste of leaf and petiole singly or in combination	against bronchitis, indigestion, and as an antidote to poison against bronchitis, indigestion, and as antidote to poison	^268^
Vettrilai	Villupuram district of Tamil Nadu, India	fresh leaves chewed or immersed with sesame oil, then warmed with flame	for digestive, stimulative, carminative, aphrodisiac, applied for headaches and lactogogue	^270^
Eman	Bulu and inland Kaulong of Papua New Guinea	pulped leaves used topically	to cure swollen limbs	^262^
Vertrilai	Kalrayan Hills, Eastern Ghats, Tamil Nadu	leaves	digestive problem	^255^
paan	tribal and native people of Madhupur forest area, Bangladesh	decoction of leaves, leaf juice	nerve pain. for joint pain, cough, and oedema	^250^
Paan	Rabha community of Mataikhar reserve forest, Kamrup district, Assam, India	leaf	castor oil is smeared on leaves, warmed and applied to affected areas for arthritis, cold, cough and headache	^61^
Base, sirih,	Bali, Indonesia	decoction of leave	body odour, and for treating diarrhoea, sore throat, skin allergies, fluor albus	^57^
patiwa	Chungtia village, Nagaland, India	leaf paste used topically or chewed with lime, areca nut and tobacco	cure cuts and wounds, to treat dental caries	^253^
pan	Parsa district, Nepal	leaf chewing, leaf juice mixed with hot water, honey or milk	mild stimulant, cure worm, remedy for bad breath and provides mouth refreshment, improve digestion, strengthen teeth and gums, palate cleaner, treatment of nervous pains and nervous exhaustion, ease urination, analgesic, reduce cough and cold	^64^
Ikmo	Sambal‐Bolinao of Pangasinan, Philippines	leaves heat with oil and salt	rub on the body the body of jaundice patient	^249^
betle	Tobelo Dalam tribe in Aketajawe Lolobata National Park Area	leaves boil with water and taken orally	postpartum pain	
Sirih/ betle	Southern slope of Mount Merapi, Yogyakarta, Indonesia	leaf	relative cough	^257^

## 8 PHYTOCHEMICAL PROFILE

**FIGURE 3:**
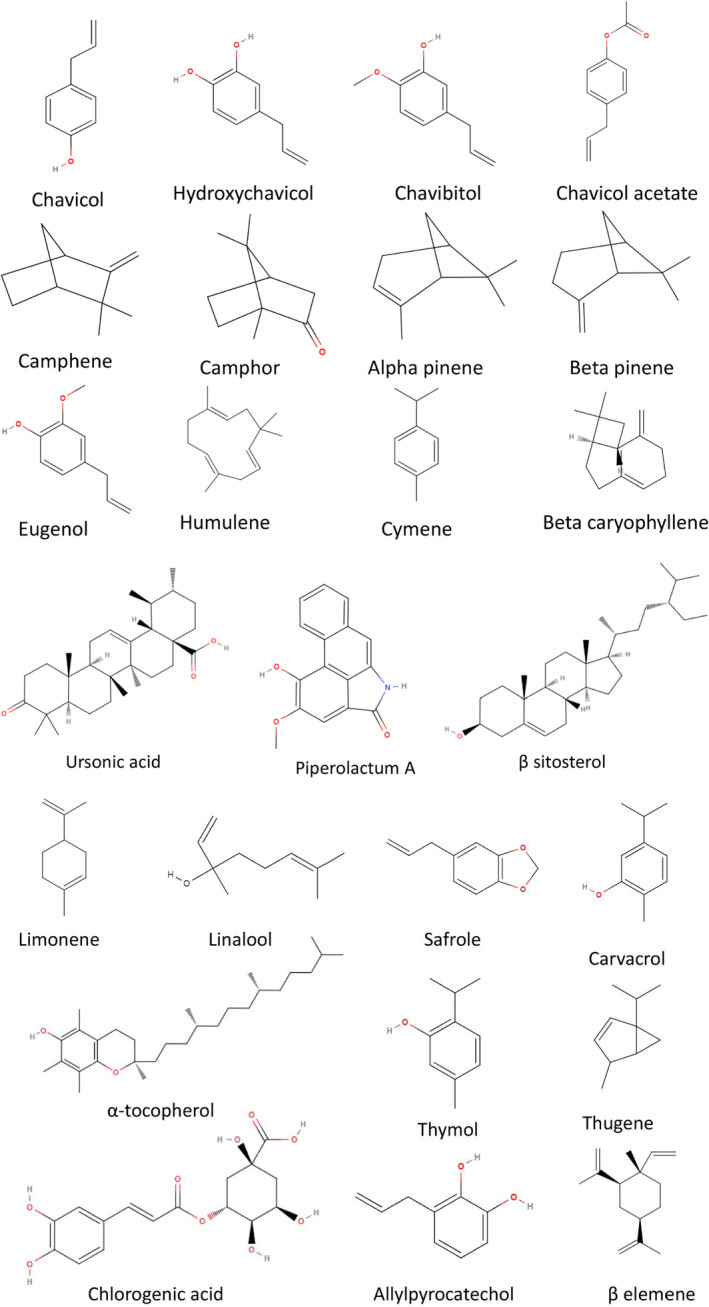
Chemical structures of some phytochemicals reported from the species

*Piper betle* is one of the extensively investigated plants for its various phytochemical constituents present in it, and the study revealed that the plant contains a wide range of phytochemicals that are biologically active. Compound concentrations depend on the different varieties of the plant, season, climate and may geographical location and also might be influenced by various factors such as soil, humidity, agronomic practices, rainfall, season and type of plant.^65^ The main phytochemical constituents of the essential oil of the betel leaf are mainly phenols and terpenes.^66^ The phenol content varies by gender, total phenols are three times higher in male plants, and the thiocyanate content is two times higher compared to female plans. Leaf quality is basically dependent on the phenol content; more phenol content comes with better leaf quality.^67^ The typical pungent aroma of the betel leaves is the result of the phenols present in them. Preliminary photochemical studies of aqueous and methanol extracts of betel leaves revealed the presence of alkaloids, flavonoids, tannins, sterols, phenols, glycosides, saponins and terpenoids.^68^ Syahidah et al. also identified alkaloids, phenols, flavonoids, saponins, steroids, tannins, terpenoids and glycosides from qualitative analysis of the methanolic extract of the betel leaves.^175^ Leaves also contain bitter compounds (0.7–2.6%).^2^ Terpenoids and their acetates, including cadinene, 1,8‐cineole, chavicol, chavibetol, safrole, camphene, limonene, caryophyllene, pinene, carvacrol, allylpyrocatechol and eugenol, are present in *P*.* betle* as the main phenols.^2^, ^69^ A recent work with the leaves was found to contain starch, diastases, sugars (0.8 to 1.8%) and an essential oil in an amount of 4.2%.^70^ The presence of tannins and steroidal components was revealed by phytochemical investigation on leaves.^71^ The main components of betel leaf oil are safrole (48.7%), chavibetol acetate (12.5%) allylpyrocate choldiacetate (34.0%), along with ρ–cymene, 4‐terpinol, eugenol, β‐caryophyllene.^72^ There are two sesquiterpenes, cadinene and caryophyllene and safrole (52.7%), eugenyl acetate (5.8%), allylpyrocatecholdiacetate (15.4%) and eugenol (6.4%) are also reported as the main elements of the essential oil of the *P*.* betle* leaf from Sri Lanka.^67^ The leaves were also found to produce an alkaloid, namely arakene, which possesses properties similar to those of cocaine. The chemical compositions of essential oil differ in different parts: leaf, stem, stalk and root contain safrole, while fruits contain β ‐phellandrene. Younger leaves of betel contain more amount of essential oil.^73^ Phytochemical analysis of two varieties of betel leaves, Kamarvetrilai and Kumbakonamvetrilai, confirmed cardiac glycosides, acids and steroids along with tannins, saponins and flavonoids.^74^ In another experiment, four cultivars of *P*.* betle*—Banarasi, Calcutta, Kammar and Kumbakonam—showed positive results in tannin, flavonoid and terpenoid tests,plobatannins found in the Banarasi cultivar, Banarasi and Kammar gave positive results for saponins,cardiac glycosides found in the Banarasi and Kumbakonam cultivars.^75^ Pipercerebrosides A and B are two new sphingolipids isolated and identified by NMR (Nuclear magnetic resonance) spectroscopy of betel leaf extract.^76^ GC‐MS (Gas chromatography–mass spectrometry) studies identified all compounds that can be divided as monoterpene (α‐thujene, α‐pinene, camphene, sabinene, myrcene, β‐phellandrene, α‐terpinene, (e)‐β‐ocimene, 1,8‐cineole/eucalyptol, γ‐terpinene, terpinolene, linalool, terpinen‐4‐ol, α‐terpineol), sesquiterpenes (δ‐elemene, α‐copaene, β‐copaene, β‐elemene, e‐β‐caryophyllene, γ‐elemene, β‐selinene, aromadendrene, α‐humulene, germacrene d, α‐selinene, γ‐muurolene, bicyclogermacrene, α‐muurolene, cis‐β guaiene, δ‐cadinene, palustrol, spathulenol, caryophyllene oxide, globulol, viridiflorol, cubenol, α‐cadinol), and phenylpropane (estragole/methyl chavicol, chavicol, anethole/isoestragole, safrole, chavicol, acetate, eugenol, methyl eugenol, eugenol acetate).^77^ Betel vine also contains dotriacontanoic acid, stearic acid, piperlonguminine, hentriacontane, n‐triacontanol, pentatriacontane, triotnacontane, isoeugenol, allylpyrocatecholdiacetate, α‐pinene, β‐sitosteryl palmitate, 1, 8‐cineol, ursolic acid, β‐sitosterol, β‐pinene, sitosterol, ursolic acid 3β‐acetate and stigmasterol. Betel roots possess ursonic acid, piperlonguminine, stearic acid, β‐sitosteryl palmitate, β‐sitosterol, 3β‐acetyl ursolic acid, 4‐allyl resorcinol, aristololactam A II and stigmast‐4‐en‐3, 6‐dione. The betel stems were found to have stigmast‐4‐en‐3, piperine, piperlonguminine, piperdardine, dehydropipernonaline, guineensine, 6‐dione, aristololactam A‐II, pellitorine, 4‐allyl resorcinol, syringaresinol‐O‐β‐D‐glucopyranoside, N‐isobutyl‐2E,4E‐dodecadienamide, pinoresinol, piperolein‐B, cepharadione A, dotriacontanoic acid, β‐daucosterol, tritriacontane, β‐sitosterol, α‐ethyl glucoside (2E,4E)‐N‐isobutyl‐7‐(3’,4'‐methylenedioxyphenyl)‐2,4‐heptadienamide, 23‐hydroxyursan‐12‐en‐28‐oic acid, (2S)‐4'‐hydroxy‐ 2,3‐dihydrofl avonone‐7‐O‐β‐D‐glucoside and β‐sitosterol‐3‐O‐β‐D‐glucoside‐6'‐O‐palmitate.^78^, ^79^, ^80^, ^81^ Gas chromatography mass spectrometry (GC–MS) analysis of fresh and cured leaves of the essential oil of *P*.* betle* var Bangla fresh and cured leaves revealed a total of thirty‐three phytochemicals and a total of thirty volatile components, respectively, with high abundance of estragole, eugenol, linalool, anethole, α‐copaene, chavicol and caryophyllene.^1^ Very recently, Islam et al. studied volatile oils from five varieties of betel such as Bangla, Misti, Khasi, Sanchi and Bari and found a total of 101 volatile oil compounds, which are much higher in number than previous reports with 50 compounds identified for the first time.^82^ Table 2 represents the phytochemical constituents of *P*.* betle*. Figure 3 represents the chemical structures of some phytochemicals reported from the species.

**TABLE 2:** Phytochemical constituents of *P*.* betle*

Plant part/Extract/Essential oil	Techniques	Chemical compounds	References
Aqueous extract of leaves	GC/MS	2,3‐bis(hydroxy)propyl ester, 2‐monopalmitin, α‐hydroxy, alpha‐hydroxyphenyl, benzeneacetic acid, benzeneacetic acid, hexadecanamide, hexadecanoic acid, hexadecanoic acid, hydroxychavicol, myristic acid, octadecanoic acid, octadecanoic acid	^258^
Essential oil from leaves	GC/MS	4‐allyl‐1,2‐diacetoxybenzene, acetyleugenol, bicyclo(4.1.0)hept‐3‐en‐ camphene, chavicol, cis‐ocimene, cyclohexene,4‐methyl‐decanal, eugenol, germacrene B, germacrene D, globulol, ledene, linalyl acetate, l‐limonene, methyl‐eugenol, phenyl acetylaldehyde, t‐caryophyllene, t‐ocimene, undecanal, α –humulene, α‐pinene, β‐elemene, β‐myrcene, γ‐cadinene, γ‐ionene, γ‐muurolene	^261^
Leaf extract	DART‐MS	chavicol, allylpyrocatechol, chavibetol, phenyl alanine, chavicol acetate, allylpyrocatechol acetate, chavibetol acetate, allylpyrocatechol, diacetate	^244^
Acetone extract and different fractions of leaf	UV/VIS/NIR, NMR, HR‐ESI‐MS, GC/MS	Sphingolipids ‐ pipercerebroside A pipercerebroside B	^76^
Volatile oil from leaves	GC/MS	β‐ caryophyllene, α‐farnesene, α‐humulene, germacrene b, germacrene d	^260^
Hexane extract of leaves	GC/MS	2,3‐dihydro‐3,5‐dihydroxy‐6‐methyl‐4h‐pyran‐4‐one, phellandrene, α‐terpinene, p‐cymene, sabinene, ɣ‐terpinene, o‐guaiacol, linalool, tujene, terpine‐1‐ol, terpine‐4‐ol, α‐terpineol, safrole, eugenol, isoeugenol, α‐copaene, β –bourbonene, methyleugenol, β –caryophyllene, β –cubebene, ɣ‐cadinene, α‐humulene, β‐selinene, α‐selinene, caryophyllene oxide, camphene, germacrene b, longifolene, phytol	^245^
Ethanol extract of leaves	GC/MS	heptafluorobutyrate, ethyl diazoacetate, 4‐(2‐propenyl)phenol, 3‐fluoro‐2‐propynenitrite, eugenol, tris(trifluoromethyl)phosphine	
Aqueous and ethanol extracts of leaves	GC/MS	*amino acid*: alanine, valine, isoleucine, proline *fatty acids*: palmitic acid, linoleic acid, linolenic acid, oleic acid, stearic acid, palmitic acid derivatives *sterols*: cholesterol, cholesterol derivatives, stigmasterol, β ‐sitosterol	^259^
Ethanol extract of leaves	GC/MS	1‐phenylpropene‐3,3‐diol diacetate, eugenol, 4‐chromanol, 4‐allyl‐1,2‐diacetoxybenzene, hydroxychavicol (1‐allyl‐3, 4‐dihydroxybenzene)	^256^
Chloroform extract of leaves	1D NMR, 2D NMR, ESI‐MS, FT‐IR and HR‐ESI‐MS	s 1‐n‐dodecanyloxy resorcinol (H1) and desmethylenesqualenyl deoxy‐cepharadione‐A	^243^
Ultrasound‐assisted extract of leaves	GC/MS	hydroxychavicol, eugenol, isoeugenol, and 4‐allyl‐1,2‐diacetoxybenzene	240, 241
Leaf aqueous extract of varieties bangla, bagerhati, manikdanga, meetha, kalibangla, chhaanchi, ghanagete and haldi	GC/MS	*amino acids*: l‐glutamic acid (dehydrated), l‐pyroglutamic acid, l‐tryptophan, *organic acids*: citric acid, 3,4‐dihydroxyphenylacetic acid, fumaric acid, gluconic acid, gluconic acid lactone, glyceric acid, glycolic acid, 3‐hydroxy‐3‐methylglutaric acid, 4‐hydroxyphenylacetic acid, isocitric acid, l‐(+) lactic acid, maleic acid, malic acid, malonic acid, nicotinic acid, oxalic acid, 3‐phenyllactic acid, ribonic acid‐gamma‐lactone, succinic acid, *sugars*: methyl‐β‐d‐galactopyranoside, isopropyl‐β‐d‐1‐thiogalactopyranoside, phenyl‐β‐glucopyranoside, sucrose, raffinose, d‐(+)trehalose, *sugar alcohols*: arabitol, galactinol, glycerol, d‐mannitol, d‐sorbitol *fatty acids*: lauric acid, myristic acid, palmitic acid, stearic acid, *phenols*: o‐acetylsalicylic acid, p‐anisic acid, benzene‐1,2,4‐triol, caffeic acid, chlorogenic acid, chrysin, cinnamic acid, coniferyl alcohol 3,4‐dihydroxybenzoic acid, ferulic acid, gentisic acid, hydroquinone, 2‐hydroxyacetophenone, 4‐hydroxybenzoic acid, hydroxychavicol, 3‐hydroxycinnamic acid, 4‐hydroxycinnamic acid, 4‐hydroxy‐3‐methoxybenzoic acid, 2‐(4‐hydroxyphenyl)ethanol, 4‐(2‐hydroxyethyl)phenol (tyrosol), 3‐(4‐hydroxyphenyl)propionic acid(synonym: hydro‐p‐coumaric acid), piceatannol, shikimic acid, quinic acid, terpenoid, loganin other organic compounds: adenosine, (‐)‐epinephrine, indole‐3‐acetamide, porphine	^77^
Leaves volatile compound from five varieties (bangla, khasia, misti, sanchi, bari)	SDE‐ GC/MS	(E)‐2‐hexenyl acetate, (E)‐cadina‐1,4‐diene, (E)‐cadinol, (E)‐calamenene, (E)‐ocimene, (E)‐Verbenol, (Z)22‐pentenyl acetate, (Z)‐3‐hexenyl‐1‐acetate, (Z)‐a‐ bergamotene, (Z)‐a‐bisabilene, (Z)‐aabinene hydrate, 4‐d‐carene, 1‐hexanol, 1‐H‐indol, 1‐nor‐bourbonanonee c, 2,3‐butanediyl diacetate, 2‐ethylfuran, 2‐hexen‐1‐ol, 2‐hexenal, 2‐penten‐1‐ol, 2‐pentylfuran, 2‐phenylethyl acetate, 3‐hexen‐1‐ol, 3‐hexenal, 4‐allylphenyl acetate, 4‐vinyl guaiacol, 9‐epi‐b‐caryophyllene, α‐amorphene, α ‐curcumene, α ‐guaiene, α ‐humulene, α ‐muurolene, α ‐muurolol, α ‐nerolidola c, α ‐pinene, aromadendrene, α ‐terpineol, α ‐thujene, β‐(z)‐bergamotene, β ‐bisabolol, β ‐caryophyllene, β ‐cyclocitral, β ‐elemene, benzaldehyde, benzyl acetate, β ‐pinene, β ‐selinene, β ‐spathulenol, cadalene, cadin‐4‐en‐10‐ol, cadina‐1(6),4‐diene, c‐amorphene, camphene, caryophyllene oxide, ɣ‐elemene, chavicol, ɣ ‐muurolene, ɣ ‐terpinene, cubebol, δ‐cadinene, decanal, dehydrocineole, dimethylallyl acetate, epicubenol, eremophilene, estragole, eugenol, eugenyl acetate, farnesyl acetate, farnesyl acetone, furfuraldehyde, guaiac acetate, hexanal, humulene epoxide ii, isogermacrene d, isophytol, limonene, linalool, linalool oxide acetate, methyl heptenone, methyl salicylate, methyleugenol, n‐butyl benzene, n‐decyl acetate, n‐dodecanal, n‐hexyl acetate, nonanal, octadecanol acetate, oxophorone, p‐cymen‐8‐ol, p‐cymene, phenyl acetaldehyde, phenylethyl alcohol, phytone, pogostol, salvial‐4(14)‐en‐1‐one, sesquisabinene, terpinen‐4‐ol, tetradecanal, undecan‐2‐one, valencene	^82^
*P*.* betle* var. *haldia* and *maghai*	1D NMR, 2D NMR, ESI‐MS, FT‐IR and HR‐ESI‐MS	1‐n‐decanoyl hydroxybenzoic acid/1‐n‐decanoyl phenol and 3‐butylphenol	^242^

## 9 PHARMACOLOGICAL ACTIVITIES

The following section summarizes the various pharmacological attributes of *P*.* betle* (Table 3).

**TABLE 3:** Pharmacological activities of *P*.* betle*

Activity	Part used (compound)	Design	Model	Effects	Reference
Anticancer/Anti tumour/Anti proliferative activity	aqueous extract of leaves	tumour inhibition assay	benzo(a)pyrene‐induced tumours in hamster buccal pouches	both short‐term and long‐term studies, expressed complete or partial suppression of tumour	^232^
	aqueous extract of the leaves	tumour inhibition assay	dimethylbenz[a]anthracene (DMBA)‐induced mammary carcinogenesis in Holtzman rats	higher doses of the extract inhibited the emergence of tumours	^91^
	alcoholic extract (eugenol, hydroxychavicol, β‐carotene and α‐tocopherol)	anticarcinogenicity studies	benzo[a]pyrene‐induced foestomach neoplasia in male Swiss mice	decreased number of papillomas per animal (by β‐carotene and α –tocopherol)	^92^
	leaf extract (hydroxychavicol)	tumour suppression assay	4‐(N‐nitrosomethylamino)‐1‐(3‐pyridyl)‐1‐butanone induced mutagenesis and tumorigenesis in mice	reduced the tumorigenic effects by 25%	^93^
	leaf extract (beta‐carotene, alpha‐tocopherol, eugenol and hydroxychavicol)	tumour inhibition assay by topical administration and intraperitoneal injection	7,12‐dimethylbenz(a)anthracene (DMBA) induced skin tumours in mice	inhibition of tumour formation by 83–94%; eugenol showed minimal protection	^72^
	leaf extract (β‐carotene and α‐tocopherol) or combined with turmeric	tumour inhibition assay	methyl (acetoxymethyl) nitrosamine‐induced hamster oral carcinogenesis	inhibition of tumour incidence, reduction of tumour burden, extension of the tumour latency period, and regression of established, frank tumours	^94^
	ethanol extract of leaves	morphological studies, MMTV‐RT assay	mammary tumour virus‐induced and 7–12‐dimethylbenz(a)anthracene‐induced rodent mammary tumours	reduced tumour incidence by 75%, tumour burden by >90%	^95^
	methanol extract	inhibitory assay of Epstein‐Barr virus (EBV) activation	Raji cells induced by 12‐O‐hexadecanoylphorbol‐13‐acetate	antitumour activity in terms of cancer chemoprevention	^96^
	leaf aqueous extract	*in vitro*neutral red cytotoxicity assay	KB and HeLa cell line	cytotoxicity on the KB cell line 29.5 ± 0.3 µg/ml, no effect towards HeLa cell line	^97^
	leaf ethanol extract	*in vitro*MTS assay	breast cancer cell line T47D	inhibit cell proliferation with IC_50_ 55.2 µg/ml	^101^
	water, methanol, ethyl acetate and hexane extracts of leaves	*in vitro*MTT assay	breast cancer cell line, MCF‐7	the ethyl acetate and hexane extracts showed dose‐dependent inhibitory effects with IC_50_ values of 65.00 and 163.30 μg/ml	^99^
	methanol extract of leaves (hydroxychavicol)	*in vivo*tumour growth and bioluminescent imaging, MTT assay	prostate cancer PC‐3 cells implanted in male BALB/c nude mice	oral feeding effective in tumour growth inhibition	^100^
	aqueous extract of root	*in vitro*MTS assay	T47D human ductal breast epithelial tumour cell line	reduce 2.8% cell proliferation, induce apoptosis 9.45%	^98^
	ethanol extract of leaves	inhibiting proliferation cells and by SubG1 flow cytometry	cervical cancer cells HeLa	growth inhibition with IC50 value 7.13 µg/ml, apoptotic activity with IC50 value 12.5 µg/ml (95.87%)	^102^
	leaf extract (hydroxychavicol)	*in vitro*MTT assay, *in vivo* histopathologic and immunohistochemical analysis	androgen‐independent human prostate cancer cells, PC‐3, DU145, C4‐2, and 22Rv1; BALB/c nude mice y injected with PC‐3‐luc cells	sensitivity was 22Rv1> C4‐2> PC‐3> DU14; inhibits growth and proliferation via ROS generation and caspase dependant pathway in P‐3 cells	^9^
	leaf acetone extract	*in vitro*MTT assay	lung cancer cell line (A549)	cell toxicity‐ 88.7%, cell death 11.4%	^103^
	petroleum ether, ethyl acetate, aqueous, and ethanol extract of leaves	*in vivo*tumour growth study	B16F10 melanoma in C57BL/6 Mice	ethyl acetate extract showed the highest dose dependant reduction in tumour size	^104^
	crude ethanol extract	cytotoxicity and suppression of cell migration determination, SRB wound healing assays, evaluation of transdermal patches	human breast cancer MCF‐7 cells	cytotoxicity with an IC_50_ of 114.3 µg/ml, suppressed cell migration at a dose of 25 µg/ml, developed a transdermal patch containing 0.03% of the extract	^105^
	leaf extract (hydroxychavicol)	colony formation assay, Annexin‐V/PI assay, cell cycle and cell death analysis, comet assay, scratch assay, Transwell migration and invasion assays	MIA PaCa‐2, PANC‐1, L929, INT407, NIH‐3T3, Vero and HEK293 cells	inhibits proliferation and epithelial‐mesenchymal transition, migration and invasion of cells, induces DNA damage, mitotic catastrophe and apoptosis	^106^
Analgesic, anti‐inflammatory, antinocepective activity	hot and cold water extract of leaves	tail flick, hot plate, and formalin tests	cross bred albino mice	hot plate and formalin tests were most effective mediated via opioid mechanisms	^110^
	ethanol extract of leaves	Freund's adjuvant‐induced model of arthritis	rat	anti‐inflammatory and anti‐arthritic effect by down regulation nitric oxide	^111^
	water, ethanol, ethyl acetate and hexane extract of leaves	hyaluronidase (HYA), xanthine oxidase (XOD), and lipoxygenase (LOX) inhibition assay	*in vitro*assay	all extracts showed significant inhibition activity	^112^
	ethanol extract of leaves	carrageenan‐induced hind paw oedema model, hot plate, writhing, and formalin tests	Swiss albino mice and Wistar rats	inhibit paw oedema, also reduced writhing and number of lickings in dose dependant manner	^113^
	ethanol extract of leaves	acetic acid induced writhing test	Swiss Albino mice	reduced writhing response via modulation of the arachidonic acid pathway	^114^
	aqueous extract of leaves	eddy hot plate and heat conduction method	Mice and rats	significant analgesic activity, dose‐dependent increase in latency period	^115^
	methanol extract of leaves (9 varieties)	‐	LPS induced RAW 264.7 cell line	five varieties showed anti‐inflammatory activity	^116^
	Betle leaf essential oil	detection of MMP‐2 and MMP9 using Gelatin Zymography	*In vitro*assay	85% anti‐inflammatory activity	^117^
Antidepressant	ethanol extract of leaves	forced swim test and tail suspension test	Swiss albino mice	reduction in the duration of immobility compared to imipramine	^121^
	hydroalcoholic extract	forced swim test and tail suspension test	Swiss albino mice	reduced the immobility time	^122^
	volatile oil	forced swim method	albino mice	reduced immobility than standard fluoxetine	^252^
Anti axiety	hydroalcoholic extract	light/dark exploration test and elevated plus maze test	Swiss albino mice	gradual increase in the dose of extract showed improvement of anxiety	^122^
Anti stress	ethanol extract of leaves	behavioural study, luciferase reporter gene assay, melatonin estimation, gene expression study	dexamethasone (DEX) induced stress in zebrafish larvae	improved behavioural and gene expression level similar to the positive control melatonin	^124^
Anticholinesterase activity	methanol extract (hydroxychavicol and chlorogenic acid)	bio‐autographic method	‐	AChE and BChE inhibition (IC50) are 21.23 ± 0.33 μg/ml and 45.55 ± 1.89 μg/ml, respectively	^126^
	aqueous and ethanol extract, hydroxychavicol	3‐(4,5‐dimethylthiazol‐2‐yl)‐2,5‐diphenyltetrazolium bromide reduction and lactate dehydrogenase leakage	human neuroblastoma cells (SH‐SY5Y)	activity against both AChE and BChE, cytotoxic to human neuroblastoma cells at concentrations higher than 500 μg/ml	^125^
Alzheimer's disease	aqueous extract of leaves	Morris water maze test and Passive avoidance test	aluminium chloride (AlCl_3_) induced Alzheimer's disease in Wistar rats	reduced mean escape latency period, improved retention of spatial memory	^127^
Nootropic effect	hydroalcoholic extract	visual observation	Swiss male albino mice	increase in discrimination index	^128^
	aqueous extract of leaves	Y‐maze Test	Scopolamine induced amnesia in albino rats	reversal effect against amnesia with a momentous decrease in retention latency, and a major decrease in inflexion ratio	^129^
Antioxidant activity	inflorescence extract	H_2_O_2_, superoxide, hydroxyl radical scavenging assay	*in vitro*assay	free radical scavenging with 50% inhibitory concentration	^213^
	aqueous extract of leaves of Kauri variety, Ghanagete, Bagerhati (chevibetol, allylpyrocatechol)	DPPH, superoxide radical scavenging activity in a riboflavin/light/NBT system, hydroxyl radical scavenging activity and inhibition of lipid peroxidation induced by FeSO4 in egg yolk	*in vitro*assay	antioxidant capacity in order of Kauri>Ghanagete>Bagerhati	^132^
	cold ethanol extract, hot water extract of leaves and essential oil	DPPH free radical scavenging assay	*in vitro*assay	free radical scavenging effects decreased in the order Cold ethanol extract >essential oil >hot water extract	^133^
	ethyl acetate, methanol, water, petroleum ether extract of leaves	DPPH assay, TBARS assay, hydroxyl radical scavenging assay	*in vitro*assay	significant antioxidant activity by all extracts	^32^
	aqueous extract of leaves	DPPH radical scavenging assay	T47D human ductal breast epithelial tumour cell line	83% antioxidant activity	^98^
	ethanol extract of leaves	superoxide dismutase activity assay	HeLa cell line	scavenged more than 50% free radical	^102^
	methanol, ethanol, acetone, ethyl acetate, and distilled water extract of leaves (Banarasi, safeda, Calcutta, Cuttack, Desibagla, Maharashtra and Sofia varieties)	DPPH, ABTS radical scavenging activity FRAP, and photochemiluminescence assay	*in vitro*assay	FRAP and ABTS assay of the Banarasi and safeda varieties and the photochemiluminescence assay for the Calcutta variety showed the highest antioxidant activity	^134^
	crude ethanol extract of leaves	DPPH radical scavenging assay	human breast cancer MCF‐7 cells	antioxidant activity with (IC_50_) of 30.0 ± 0.1 µg/ml	^105^
	ethanol, ethyl acetate, hexane +petroleum ether and aqueous extract of leaves	DPPH scavenging assay, reducing power activity, hydrogen peroxide scavenging assay	*in vitro*assay	all extracts showed good antioxidant properties in all assays in concentration dependant manner	^135^
	ethanol extract of leaves	oxygen radical absorbance capacity (ORAC) Assay	*in vitro*assay	potential free radical scavenging activity	^136^
	leaf methanol extract	nitric oxide, hydroxyl radical and reducing power assay, ferric ion RPA method	*in vitro*assay	fewer antioxidant activity compared to eugenol	^137^
Hepatoprotective activity	aqueous extract or leaf	biochemical estimation	ethanol‐induced hepatotoxic and oxidative damage in Wistar rat	decreased aspartate aminotransferase (AST), alanine aminotransferase (ALT), thiobarbituric acid reactive substances (TBARS), and lipid hydroperoxides; improved non‐enzymatic antioxidants and free radical detoxifying enzymes	^138^
	aqueous extract of leaf	biochemical and histopathological study	Wistar rats liver fibrosis induced with carbon tetrachloride (CCl_4)_ and corn oil	inhibited AST and ALT activities; attenuated total glutathione S‐transferase activity (GST); enhanced superoxide dismutase (SOD) and catalase (CAT) activities; attenuated liver fibrosis, decreased expression of α‐smooth muscle actin (α‐SMA), induced expression of active matrix metalloproteinase‐2 (MMP2), and inhibited TIMP2 level	^140^
	leaf extract	‐	oxidative stress‐induced D‐galactosamine intoxication in Wistar rat	improved antioxidants –lipid hydroperoxidase, SOD, GSH peroxidise, vitamin C, vitamin E, GSH; decreased TBARS, hydroperoxidase and liver marker enzymes AST, ALT, alkaline phosphate (ALP), gamma glutamyltranspeptidase (GGTP)	^139^
	ethanol extract of leaves	acute toxicity, serum hepatic enzyme level and antioxidant enzyme level study	liver damage in Wistar rats induced with CCl_4_	reduced serum glutamate oxaloacetate transaminase (SGOT), serum glutamate pyruvate transaminase (SGPT), (ALP), acid phosphatase, lipid peroxidation; improved catalase, SOD, glutathione (GSH) in liver	^141^
	ethanol extract of leaves	Biochemical and histopathological assay	cadmium chloride‐induced liver dysfunction in Wister rat	altered elevated level of serum AST, serum ALT, ALP, lactate dehydrogenase (LDH), gamma‐glutamyl transpeptidase (GGTP), bilirubin; oxidative stress markers TBARS, lipid hydroperoxide (LOOH), protein carbonyl, conjugated dienes, reduced SOD, CAT, GST vitamin C and vitamin E in the liver	^142^
	ethanol extract of betle leaves	biochemical and histochemical studies	methotrexate‐induced hepatotoxicity in Sprague‐Dawley rats	reduced ALT, AST, ALP level; reduced central vein dilatation, leukocyte infiltration, normalized hepatocellular architecture, reduced LPO, increased depleted GSH level and SOD, CAT, and GPx	^143^
Anti ulcer activity	ethanolic extract of leaves	assay of MDA, oxidatively damaged protein, SOD, CAT, hexosamine, mucus, and free radical scavenging activity	indomethacin‐induced gastric lesion in Sprague‐Dawley rats	Significant protection against gastric lesions, increased SOD and CAT activity, increased mucus, hexosamine and total thiol group content, reduced oxidative damaged protein and peroxidized lipid level, increased free radical scavenging action	^144^
	ethanol extract of leaves	histochemical investigation	NSAID‐induced ulcer in Charles Foster rats	increased antioxidative factors, mucus, and total gastric tissue sulfhydryl group	^145^
	leaf ethanol extract (isolated allylpyrocatechol)	histological and biochemical investigation	indomethacin‐induced stomach ulceration in Sprague‐Dawley rats	reduced the ulcer index by 93.4%, accelerated ulcer healing, improved the mucin content of gastric tissues, showed normal malondialdehyde (MDA) and protein level, increased the SOD and CAT	146, 147
	hydroalcoholic extract of leaves	acute toxicity test and ulcer index study	HCl‐ethanol, acute stress, and pylorus ligation models in Wistar rats and Swiss albino mice	decreased ulcer index, increased gastric pH, and decreased gastric fluid volume	
	hot and cold aqueous extract of leaves	effects on mucus content of the gastric mucosa, total and free acidity, volume and pH of the gastric juice study	Ethanol‐induced crossbreed albino rats	increased the mucus content adhering to the wall of the gastric mucosa and inhibited the volume of gastric acid	^31^
Antihyperglycaemic activity	leaf suspension	plasma levels of glucose and glycosylated haemoglobin, activities of liver hexokinase and gluconeogenic enzymes assay	streptozotocin diabetic albino Wistar rats	reduction in blood glucose and glycosylated haemoglobin, decreased activities of liver glucose‐6‐phosphatase and fructose‐1,6‐bisphosphatase, increased liver hexokinase in a dose dependant manner	^149^
	methanol extract of leaf (Bangla variety)	biochemical study, spectroscopic study	*in vitro*BSA‐glucose model	inhibit glucose‐induced glycation, thiol group modification and carbonyl formation	^150^
	ethanol extract of leaves	aldose reductase assay	*in vitro*assay	inhibition of human recombinant aldose reductase (HRAR) contradiction	^136^
Antihyperlipidaemic activity	aqueous extract of leaves	‐	brain of ethanol administered Wistar rats	co‐administration resulted in reduction of lipid levels (free fatty acids, cholesterol, and phospholipids) and lipid peroxidation markers	^151^
	methanol extract of leaves		fat diet induced hyperlipidemia in Wistar rat	depletion in total cholesterol (TC), triglycerides (TG), low‐density lipoprotein (LDL) and very low‐density lipoprotein‐cholesterol (VLDL) activity levels in serum	^152^
Anti‐ atherogenic activity	ethanol extract of leaves	‐	Triton WR‐1339‐induced hypercholesterolemia in Wistar rat	ameliorated hypercholesterolemia induced high level of TC, TG, LDL and VLDL and low level of enzymatic and non‐enzymatic antioxidants	^153^
	ethanol extract of leaves (eugenol)	Biochemical and histopathological study	Atherogenic diet fed Wister rat	lower levels of TC, TG, LDL and VLDL cholesterol in serum and liver tissue; low aspartate aminotransferase, alanine aminotransferase, alkaline phosphatase, lactate dehydrogenase, and lipid‐metabolizing enzymes in serum; low enzymatic antioxidant; higher malondialdehyde in haemolysate and hepatic tissue	^154^
Cardioprotective activity	hydroalcoholic extract of leaves	surgery for haemodynamic, measurement of left ventricular function measurement, biochemical study	rat with isoproterenol (ISP)‐induced myocardial infarction	modulated haemodynamic systolic, diastolic, mean arterial pressure (SAP, DAP, and MAP) and ventricular function parameters‐ contractility (+LVdP/dt), and relaxation (‐LVdP/dt), heart rate (HR), restored SOD, CAT, GSH, and GPx, reduced leakage of the CK‐MB isoenzyme and LDH, decreased lipid peroxidation in the heart	^155^
	ethyl acetate extract of leaves (eugenol)	intracellular ROS levels assay, cellular antioxidant enzyme profile, detection of apoptosis with annexin V‐PI	rat heart cell line H9c2 incubated with H_2_O_2_	cytoprotective effect against H_2_O_2_ induced oxidative stress, decreased intracellular ROS and apoptosis	^156^
Antifertility	Stalk alcoholic extract	‐	adult male and female rats and rabbits	number of pups reduced, anti‐oestrogenic property recorded, mild progestational activity in immature oestrogen‐primed rabbits with some follicle depressant type in their regressive phase	^157^
	ethyl alcohol extract of leaf stalk	sperm motility and count, fertility, biochemical study	male Swiss albino mice	reduced fertility to 0%, suppressed sperm mobility and cauda epididymal sperm count, reduced fructose content in the seminal vesicle, increased cholesterol in testes	^158^
	ethanol extract of petiole	oestrus cycle, fertility, litters per rat and oestradiol concentration, haematology and serum biochemistry study	female albino Wistar rats (Rattus norvegicus)	reduction in reproductive organ weights, oestrogen level, fertility, litter number, serum glucose concentration, acid phosphatase, SGOT and SGPT activity, increased cholesterol and ascorbic acid activity, non‐utilization of cholesterol and mobilization of ascorbic acid, irregular oestrus cycle, no change in haematological parameters	^159^
	aqueous and methanol extract of leaves	Fertility study, effect on oestrous cycle	vaginal smear of female albino Wistar rat	irregular and prolonged oestrous cycle which result in infertility	^68^
	root extract (Piperolactam A)	molecular docking, ligand binding affinity, molecular dynamics study	*in silico*study	potential contraceptive activity with high binding affinity to the oestrogen and progesterone receptor, the binding site has more hydrogen binding with receptor	^160^
	petroleum ether, ethanol, and water extract of whole plant	antifertility, reproductive outcome, anti‐implantation, abortifacient, hormonal study	adult female Wistar rat	significant antifertility, anti‐implantation and abortifacient activity, reduced level of follicle stimulating hormone (FSH), luteinizing hormone (LH), progesterone, anti‐oestrogenic activity, irregular oestrous cycle	^161^ ^,^ ^162^
Antiplatelet	n butanol extract and fractions of roots (isolated ursonic acid, 3B‐acetyl ursolic acid and B‐sitosterol)	*in vitro*study	arachidonic acid, platelet activation factor (PAF), and adenosine diphosphate (ADP)‐induced human platelet aggregation (PA)	ursonic acid, 3B‐acetyl ursolic acid, and B‐sitosterol have decreasing potency for arachidonic acid‐induced PA inhibition; ursonic acid and B‐sitosterol inhibited PAF‐induced PA, B‐sitosterol inhibited ADP‐ induced PA	^212^
	aqueous extract of inflorescence	*in vitro*assay	arachidonic acid (AA) induced and collagen‐induced rabbit platelet aggregation	inhibited platelet aggregation with IC_50_ 207 and 335 µg/ml, inhibited AA‐, collagen, and thrombin‐induced thromboxane B2 (TXB2) production by >90%	^213^
Anti‐halitosis	methanol extract and fractions of leaves and isolated compound allylpyrocatechol (APC)	MIC, Biofilm, methyl mercaptan and hydrogen sulphide volatile sulphur compound (VSC) assay	*in vitro*saliva chip model	the reduction in the VSC production by oral anaerobic bacteria due to the antimicrobial activity of APC also prevented periodontal infection	^214^
Antiallergic activity	ethanol extract of leaves	histamine and granulocyte macrophage colony‐stimulating factor (GM‐CSF); eotaxin and IL‐8 production study	*in vitro*assay	decreased histamine and GM‐CSF production and inhibited eotaxin and IL‐8 secretion	^215^
Anti‐asthmatic effect	ethanol extract	calculation of proconvulsive time	histamine aerosol induced asthma in guinea pig	significant anti‐asthmatic effect at doses of 100 mg/kg and 200 mg/kg body weight, prolonged the latent period of convulsions	^216^
Anti dermatophytic activity	ethanol extract of leaves	broth dilution, disc diffusion assay	against selected zoonotic dermatophytic fungi, viz. *Microsporum canis*, *Microsporum gypseum*, *Trichophyton mentagrophyte* and *Candida albicans*	very effective antifungal activity with IC_50_ values ranging from 110.44 to 119.00 µg/ml	^217^
Anti‐hematolytic activity	water, methanol, ethyl acetate, and petroleum ether extracts of leaves	‐	*in vitro*H_2_O_2_ treated human erythrocytes model	reduced haemolysis without any toxicity	^32^
Thyroid function	leaf aqueous extract	triiodothyronine T_3_ and thyroxine T_4_concentratiodetermination	Swiss albino male mice	higher doses decreased T_3_ and increased T_4_ concentrations, the lowest dose increased T_3_ and decreased T_4_	^218^
Immunomodulatory activity	methanol extract of leaves	lymphocyte proliferation assay, delayed type hypersensitivity reaction, determination of antibody titre	*in vitro*assay and *in vivo* assay in Swiss albino mice immunized with sheep red blood cells	immunosuppressive effect on cellular and humoral response by dose‐dependent suppression of peripheral blood lymphocyte proliferation, decreased antibody titre, increased inflammation suppression	^71^
	crude methanol and n‐hexane fraction of plant	assessment of humoral immune response, cellular immune response, flow cytometry	*in vivo*assay in Balb/c mice infected with the human lymphatic filarial parasite *Brugia malayi*	enhancement of both humoral and cell‐mediated immune responses, increased population of T cells and B cells, produced type 1 and type 2 cytokine responses	^204^
Radioprotective activity	ethanol extract of leaves	lipid peroxidation, DNA strand break, 2‐deoxyribose, superoxide scavenging, and lymphoproliferation assay	in vitro ɣ‐irradiated rat liver mitochondria and pBR322 plasmid DNA as two model	prevented ɣ‐ray induced lipid peroxidation (thiobarbituric acid reactive substrates, lipid hydroperoxide and conjugated diene), DNA strand breaks, improved hydroxyl and superoxide radical scavenging property along with its lymphoproliferative activity in a concentration‐dependent manner	^55^
Activity against acne	cream dose of ethanol extract	disc diffusion method, minimum inhibitory concentration	bacteria *Staphylococcus aureus* and *Propionibacterium acnes*	antibacterial activity with a MIC value of 4.5% and 4.0%	^219^
	noisome gel containing leaf essential oil	Franz diffusion cell	*Propionibacterium acnes*	inhibition of bacteria	^221^
	ethanol extract cream	disc diffusion method	*Propionibacterium acnes*	15% cream‐containing extract showed highest inhibition	^220^

### 9.1 Antitumour/anticancer/antiproliferative activity

One of the promising therapeutic strategy to inhibit cancer cell proliferation is to facilitate apoptosis. In cancer research, finding apoptosis‐inducing agents derived from plant sources has become popular due to the fact that existing anti‐apoptotic drugs, many of which are derived from chemical substances, often fail to combat cancer development and progression.^83^ In addition to the destruction of rapidly proliferating cancer cells, many anticancer compounds also kill normal cells in the body. Cancer can be treated with chemotherapy and/or radiotherapy, but both can cause numerous adverse health effects, and in many instances, cancer cells develop resistance to anticancer medications. However, of late few compounds obtained from natural sources, which cannot even be synthesized in the most advanced chemical synthesis laboratories, have shown great promise in the cancer treatment.^84^ ^,^ ^85^ ^,^ ^86^ ^,^ ^87^ ^,^ ^88^ ^,^ ^89^

The first report of antitumour activity of *P*.* betle* came from Rao. He studied the activity of the aqueous extract prepared from leaves in benzo(a)pyrene‐induced tumours in buccal pouches of hamsters. The result revealed that the betel leaf extract was very effective in inhibiting preneoplastic and neoplastic changes; partial and complete tumour suppression was also observed in both short‐term (10 days) and long‐term (6 months) treatment.^90^ Again, the effect of the aqueous extract of leaves on dimethyl benz (a) anthracene (DMBA)‐ induced carcinogenesis in the mammary grand of Holtzman rats was evaluated. When leaf extract was administered orally at higher doses, it showed the inhibitory result on tumour emergence.^91^ Bhide et al. investigated the result of the alcoholic extract of betel leaves and its few constituents (hydroxychavicol, α‐tocopherol, eugenol and β‐carotene) against benzo[a]pyrene‐induced neoplasia in the forestomach of Swiss male mice. The leaf extract of betel and the constituents present in it were able to decrease the number of papilloma, and the highest protection was shown by α‐tocopherol and β‐carotene.^92^ A study of the effect of leaf extracts on the carcinogenic and mutagenic actions of nitrosamines, 4‐(N‐nitrosomethylamino)‐1‐(3‐pyridyl)‐1‐butanone (NNK), which is one of the most potent chemicals specific to tobacco, was carried out in mice. The result showed that leaf extract and hydroxychavicol were able to reduce the tumour‐forming efficacy of NNK by approximately 25%, and inhibited the decrease in vitamin A levels by the induction activity of NNK in plasma and liver.^93^ Azuie et al. studied the tumour inhibition activity of the betel leaf and its constituents in 7,12‐dimethylbenz(a)anthracene (DMBA) induced skin tumours in mice and found inhibition of tumour formation by 83–84%.^72^ They also investigated oral carcinogenesis induced by methyl (acetoxymethyl) nitrosamine in hamster, extract treatment resulted in inhibition of tumour incidence, reduction of tumour burden, extension of tumour latency period, and regression of established and honest tumours, suggesting that betel can be used to develop a potential chemopreventive agent for human oral cancer.^94^ In another experiment, Bhide et al. showed that the incidence of virus‐induced and 7–12‐dimethylbenz (a) anthracene‐induced rodent mammary gland tumours can be reduced by 75% and tumour burden by >90% by the administration of the ethanol extract of the leaves.^95^ The methanol extract prepared from the leaves was able to exhibit antitumour activity in terms of cancer chemoprevention in Raji cells induced by 12‐O‐hexadecanoylphorbol‐13‐acetate.^96^ The aqueous extract of leaves and the ethanol extract of leaves in KB cell line (human epithelial carcinoma cells)^97^ and the ethanol extract in the breast cancer T47D cell line^98^ exhibited cytotoxic and antiproliferative activity with IC_50_ values of 29.5 ± 0.3 and 55.2 µg/ml, respectively. Abrahim et al. evaluated the anticancer activity of extracts of water, methanol, ethyl acetate and hexane from leaves in the MCF‐7 breast cancer cell line. Ethyl acetate and hexane extracts showed a dose‐dependent inhibitory effect with IC_50_ values of 65.00 and 163.30 μg/ml, respectively.^99^ The anticancer benefits of betel leaves and bioguided fractionation were evaluated for prostate cancer management and found that hydroxychavicol is the most potent component to inhibit tumour formation in the PC 3 cell line.^100^ In another experiment, Widowati et al. found that the aqueous extract of *P*.* betle* root can effectively reduce cell proliferation by 2.8% and induce apoptosis by 9.45% in the T47D cell line (human ductal breast epithelial tumour)^101^ ^,^ethanolic extract of leaves can inhibit the growth of HeLa cervical cancer cells with an IC_50_ value of 7.13 µg/ml and exhibit apoptotic activity with an IC_50_ value of 12.5 µg/ml (95.87%).^102^ The *in vitro* anticancer efficacy of hydroxychavicol‐containing leaf extract showed sensitivity to androgen‐independent human prostate cancer cells (22Rv1> C4‐2> PC‐3> DU14) and the activity of *P*.* betle* in BALB/c nude mice y injected with PC‐3‐luc cells by inhibiting growth and proliferation through ROS (Reactive oxygen species) generation and caspase‐dependent pathway.^9^ An experiment with the MTT assay, 88.7% cell toxicity and 11.4% cell death were observed in the lung cancer cell line (A549) applying acetone extract of betel leaves.^103^ Shah et al. studied the tumour inhibition assay of B16F10 melanoma in mice (C57BL/6) with leaves ethyl acetate, petroleum ether, aqueous and ethanol extracts. The result revealed that the ethyl acetate extract showed the highest dose‐dependent reduction in tumour size.^104^ Recently, Boontha et al. used a crude ethanolic extract of betel leaf to assess anticancer activity in human breast cancer cells (MCF‐7) and found that the extract showed cytotoxicity with an IC_50_ value of 114.3 µg/ml, suppressed cell migration at a dose of 25 µg/ml and developed a transdermal patch containing 0.03% extract.^105^ Another *in vitro* experiment with leaf extract containing hydroxychavicol in pancreatic cancer cell lines, viz. MIA PaCa‐2, PANC‐1, L929, INT407, NIH‐3T3, Vero and HEK293 cells exhibited inhibition of cell proliferation and epithelial‐to‐mesenchymal transition in cell lines, invasion and migration of cells through generalized gene repression, induced DNA damage, and also resulted in mitotic catastrophe and apoptosis through the JNK pathway and the caspase‐mediated pathway.^106^

### 9.2 Analgesic/anti‐inflammatory/antinociceptive activity

The term ‘inflammation’ refers to the complex pharmacological process of the tissues in response to harmful stimuli *viz*. damaged cells, pathogens or irritants, which is characterized by swelling, warmth, redness and pain.^107^ There has been a growing interest in developing safe and effective drugs for pain and inflammation from both academia and the pharmaceutical industry.^108^ By different types of inflammatory model tests, researchers found that food supplements could be considered as safe natural analgesics which act as adjuvant for various clinical pain and inflammation by modulation of TRPM8/TRPA1 channels and endogenous opioids signalling pathways.^109^ The antinociceptive activity of *P*.* betle* was investigated using hot and cold‐water extracts of various concentrations in tail flick test, hot plate test and formalin test models of cross‐bred albino mice. The cold extract showed higher antinociceptive activity than the hot extract via the opioid‐mediated pathway.^110^ The anti‐inflammatory efficacy of the betel leaf ethanol extract was studied in arthritic rats with a complete Freund adjuvant‐induced model. Ethanol extract was found to reveal anti‐inflammatory and anti‐arthritic activity by down‐regulating nitric oxide generation in a dose‐dependent manner compared to positive control dexamethasone.^111^ Pin et al. investigated the anti‐inflammatory activity of *P*.* betle* leaves using various solvents (ethanol, ethyl acetate, water and hexane) by *in vitro* inhibition assay of hyaluronidase (HYA), xanthine oxidase (XOD) and lipoxygenase (LOX). The extracts did not show a good inhibitory effect in the HYA assay, but showed a greater inhibition of more than 70% in the XOD and LOX assay. The order of increasing inhibitory activity of the extracts was aqueous < ethyl acetate < ethanol < hexane.^112^ In another experiment, betel leaves methanol extract was used to study anti‐inflammatory activity with the carrageenan‐induced hind paw oedema model and analgesic activity was studied using hot plate, formalin test and writhing test. Administration of the extract significantly (*p* < 0.05) reduced carrageenan‐induced paw oedema and reduced the number of acetic acid‐induced writhing and formalin‐induced licks in a dose‐dependent manner.^113^ De et al. also observed a reduced writhing response through modulation of the arachidonic acid pathway in the acetic acid‐induced writhing test on Swiss Albino mice using ethanolic extract of leaves.^114^ The analgesic effect of the betel leaf was evaluated using the heat conduction process and the hot plate method of the eddy in mice and rat models. Dose‐dependent analgesic effect was observed by increasing the latency period.^115^ *The in vitro* anti‐inflammatory effects of several varieties of *P*.* betle* leaf methanolic extracts were evaluated in the cell line (RAW 264.7) induced by *E*. *coli* lipopolysaccharide (LPS). Five varieties among the nine varieties showed significant anti‐inflammatory activity.^116^ Another experiment was carried out in which leaf essential oil was used to evaluate the anti‐inflammatory activity of *P*.* betle* using the detection of MMP‐2 (metalloproteinase‐2) and MMP‐9 (metalloproteinase‐9) using the gelatin zymography method *in vitro*. An effective anti‐inflammatory activity with 85% inhibition was observed.^117^

### 9.3 Neuropharmacological property

Numerous neurological and psychiatric disorders such as Alzheimer's disease and Parkinson's disease as well as epilepsy, migraine and essential tremors have caused severe human morbidity and mortality.^118^, ^119^, ^120^ Depression, anxiety disorders and cognitive impairment are the most common comorbid diagnoses in neurological diseases. Treatment options include medications, cognitive‐behavioural therapy, somatic interventions or electroconvulsive therapy. Although oral antidepressants have some advantages, they also present few limitations like side effects, interaction with other medications, incompatibility and inefficiency. To find a better and safer alternative treatment of neurological conditions, natural compounds of plant origin such as terpenes, alkaloids, flavonoids, lipids and phenolic acids are being studies extensively.^87^

#### 9.3.1 Antidepressant activity

The antidepressant activity of the ethanol extract of betel leaves was evaluated in Swiss albino mice using the forced swim test and the tail suspension test. Oral administration of leaf extract showed notable antidepressant activity by reducing the duration of immobility compared to imipramine‐treated control mice.^121^ Gulhane et al. in their experiment also found that the hydroalcoholic extract from betel leaves is capable of controlling depression by reducing immobility time in the tail suspension test and forced swim test, when imipramine was used as standard drug.^122^ The volatile oil obtained from the *P*.* betle* fruit also showed a significant antidepressant effect in albino mice using the forced swim method compared to the standard antidepressant drug fluoxetine.^252^

#### 9.3.2 Anti‐anxiety activity

Anxiety is characterized as being an unpleasant emotional state for which the cause cannot be identified or perceived as uncontrollable, which impairs efficiency and induces insomnia, as well as resulting in a wide range of medically unexplained symptoms.^123^ The hydroalcoholic extract of *P*.* betle* leaves was used to assess antianxiety activity in Swiss albino mice. A gradual dose‐dependent improvement was observed using the light/dark exploration test and an increase in plus compared to the control group receiving diazepam as standard in the antianxiety model.^122^

#### 9.3.3 Antistress activity

The effect of *P*.* betle* was evaluated to understand its potential role in stress‐mediated sleep disruption mediated by early exposure to life. For this study, betel leaf ethanol extract was administered under post‐fertilization stress induced by dexamethasone (DEX) in zebrafish larvae. The results showed improved levels of melatonin‐related behavioural gene expression (MT1, MT2, aanat1 and aanat2) and stress‐related gene expression (NF‐kB) similar to positive control melatonin.^124^

#### 9.3.4 Anticholinesterase activity and against Alzheimer's disease

The neurotransmitter acetylcholine is cleaved by acetylcholinesterase (AChE) and butyrylcholinesterase (BchE); therefore, inhibition of AchE and BchE is important to enhance brain activity. Alzheimer’s disease, which is a neurodegenerative disorder that causes dementia, impaired memory and impaired cognitive function in elderly people, can be managed by the application of cholinesterase inhibitor drugs. The *in vitro* anticholinesterase activity of *P*.* betle* was investigated in human neuroblastoma cells (SH‐SY5Y) by studying viability by reducing the leakage of 3‐(4,5‐dimethylthiazol‐2‐yl)‐2,5‐diphenyltetrazolium bromide and lactate dehydrogenase. Both the aqueous extract and the ethanol extract exhibited strong inhibitory activity on AchE and BchE.^125^ Dalai et al. also evaluated the inhibitory efficacy of standardized betel leaf methanol extract, containing hydroxychavicol and chlorogenic acid against AchE and BchE. Hydroxychavicol was found to have a more potent cholinergic effect than chlorogenic acid, but a combination of both (1:1) showed the highest inhibitory activity with an IC_50_ of 21.23  ±  0.33 μg/ml and 45.55 ±  1.89 μg/ml against AchE and BchE, respectively.^126^

The effects of *P*.* betle* leaf extract on memory and learning ability were evaluated in Wistar rats with aluminium chloride‐induced (AlCl_3_) Alzheimer’s disease. Two tests, the passive avoidance test and the Morris water maze test, showed that the administration of the aqueous extract of leaves reduced the mean escape latency period and improved spatial memory retention in the same way as in rivastigmine‐treated mice.^127^ These investigations suggest that *P*.* betle* could be an excellent anticholinergic agent with potential in the therapeutic management of Alzheimer's disease.

### 9.4 Nootropic effect

The hydroalcoholic extract of *P*.* betle* leaves was shown to have nootropic effect by the experiment in which the extract was administered to Swiss albino mice and the result showed an increase in discrimination index in the object recognition test.^128^ In another experiment, the nootropic effect of *P*.* betle* in scopolamine‐induced amnesia in albino rats was evaluated using the Y‐maze test and it was found that the aqueous extract of leaves can reverse the effect against amnesia with a significant decrease in retention latency, a major decrease in the inflection ratio.^129^

### 9.5 Antioxidant activity

Formation of reactive oxygen species (ROS) is one of the major markers in any disease pathology. An antioxidant acts as a protective barrier against ROS, which causes chronic and degenerative diseases.^130^ The main health problems such as cancer, cardiovascular diseases, rheumatoid arthritis, Alzheimer’s disease and other neurodegenerative disorders may be caused by the formation of free radicals. Antioxidants are very beneficial because they scavenge these free radicals and help prevent these kinds of disorders because they reduce the oxidative injury of cell proteins, carbohydrates and lipids.^131^ The extract of the inflorescence of *P*.* betle* was found to scavenge free radical (H^2^O^2^, superoxide, hydroxyl radical) with a 50% inhibitory concentration using an *in vitro* assay. 2,2‐diphenyl‐1‐picrylhydrazyl (DPPH), superoxide and hydroxyl radical scavenging activity was calculated in a riboflavin/light/NBT (9 nitro blue tetrazolium) system and inhibition of lipid peroxidation induced by FeSO_4_ using the aqueous extract of leaves of three varieties of betel in egg yolk (Kauri, Ghanagete, Bagerhati). The antioxidant capacity was observed in the order of Bagerhati < Ghanagete < Kauri.^132^ Arambewela et al., in the DPPH free radical scavenging assay, found that the free radical scavenging effects decreased in the order of cold ethanolic extract > essential oil > hot water extract of leaves.^133^ Significant antioxidant efficacy of betel leaves methanol, water, petroleum ether and ethyl acetate extracts observed using the *in vitro* DPPH assay and the TBARS (Thiobarbituric acid reactive substance assay), hydroxyl radical scavenging assay.^32^ The leaf aqueous extract in DPPH, free radical scavenging assay showed 83% antioxidant activity in the human ductal breast epithelial tumour (T47D) cell line^98^ and the ethanolic extract of leaves scavenged more than 50% free radical in the HeLa cell line using the superoxide dismutase activity assay.^102^ Jaiswal et al. performed an antioxidant assay using methanol, ethanol, acetone, ethyl acetate and distilled water extract from betel leaves (Banarasi, safeda, Calcutta, Cuttack, Desibagla, Maharashtra and Sofia varieties). The highest antioxidant activity was observed in the FRAP assay (Ferric reducing antioxidant power) and the ABTS (2,2′‐Azinobis‐(3‐Ethylbenzthiazolin‐6‐Sulfonic Acid) assay of Banarasi safeda and photochemiluminescence assay for the Calcutta variety.^134^ The DPPH radical scavenging assay in human breast cancer MCF‐7 cells using crude ethanolic extract of leaves showed antioxidant activity with (IC_50_) of 30.0 ± 0.1 µg/ml.^105^ used different solvent extracts of leaves (ethanol, ethyl acetate, hexane +petroleum ether and aqueous extract of leaves) and found potential antioxidant properties of all extracts in all assays (DPPH, reducing power activity and hydrogen peroxide scavenging assay) in a concentration‐dependent manner^135^ Ethanolic extract from leaves using the *in vitro* ORAC (oxygen radical absorbance capacity) assay^136^ and methanol extract of leaves using *in vitro* nitric oxide, hydroxyl radical and reducing power assay, ferric ion RPA (Robotic Process Automation) method^137^ showed potential antioxidant activity through free radical scavenging.

### 9.6 Hepatoprotective property

The hepatoprotective activity of *P*.* betle* was investigated using a model of ethanol‐intoxicated hepatotoxic injury in the Wistar rat. Oral administration of betel leaf ethanolic extract at a dose of 300 mg/kg bw was found to show the highest activity, namely decreased AST (aspartate aminotransferase), TBARS (thiobarbituric acid reactive substances), ALT (alanine aminotransferase) and lipid hydroperoxides; improved non‐enzymatic antioxidants such as reduced GSH (glutathione), vitamin E and vitamin C, and free radical detoxifying enzymes such as CAT (Catalase), SOD (Superoxide dismutase) and GSH peroxidase in kidney and liver of rats.^138^ Treatment with betel leaf extract improved D‐galactosamine intoxication induced by oxidative stress in Wistar rats. The extract at a dose of 200 mg/kg bw improved antioxidant levels such as lipid hydroperoxidase (LOOH), SOD, GSH, GSH peroxidise, vitamin E and vitamin C,decreased TBARS, hydroperoxidase and liver marker enzymes such as ALT, AST, alkaline phosphate (ALP) and gamma glutamyl transpeptidase (GGTP).^139^ To evaluate the hepatoprotective activity of *P*.* betle*, Young et al. induced liver fibrosis with carbon tetrachloride (CCl_4_) and corn oil in Wistar rats. The leaf aqueous extract was found to attenuate liver fibrosis by inhibiting AST and ALT activities, attenuating total glutathione S‐transferase activity (GST) and decreasing the expression of a‐smooth muscle actin (α‐SMA). The extract also enhanced the expression of active matrix metalloproteinase‐2 (MMP2) induced by SOD and CAT activities and inhibited the level of TIMP2 (Tissue inhibitor of metalloproteinases 2).^140^ Manigauha et al. also studied the effect of ethanolic extract from betel leaves against CCl_4_ induced liver damage in Wistar rats. Administration of leaf extract significantly reduced serum glutamate oxaloacetate transaminase (SGOT), serum glutamate pyruvate transaminase (SGPT), (ALP), acid phosphatase and lipid peroxidation, it also improved CAT, SOD, GSH in the liver of rats.^141^ Altered levels of elevated serum AST, ALT, ALP, lactate dehydrogenase (LDH), GGTP, bilirubin,TBARS, LOOH, protein carbonyl, conjugated dienes, reduced SOD, CAT, GST, vitamin C and vitamin E were observed when liver dysfunction induced with cadmium chloride in Wister rats treated with ethanol extract of betel leaves.^142^ Ethanol extract prepared from betel leaves was also found to mitigate methotrexate‐induced hepatotoxicity in rats (Sprague‐Dawley) by reducing ALT, AST and ALP levels. Histological studies showed that the extract reduced central vein dilation, leukocyte infiltration and normalization of the hepatocellular architecture,the extract also reduced LPO levels and increased the depleted GSH level and SOD, CAT and GPx (glutathione peroxidase) by methotrexate.^143^

### 9.7 Antiulcerogenic property

The prolonged use of non‐steroidal anti‐inflammatory drugs (NSAID) is one of the main causes of peptic ulcer, and there are other factors such as alcohol abuse and acute/chronic stress. Majumder et al. evaluated the antiulcerogenic efficacy of *P*.* betle* against gastric injury induced by indomethacin in Sprague‐Dawley rats. Oral administration of ethanol leaf extract at 200 mg / kg bw of dose for ten days showed noteworthy protection against gastric lesions, increase in SOD and CAT activity, amplification of mucus quantity, increase in hexosamine and total thiol group quantity, but reduced the amount of damaged oxidative protein and peroxidized lipid level, increased free radical proving the antiulcerogenic potential of betel by antioxidant mechanism.^144^ The authors in another experiment also found that the ethanolic extract of betel leaves can protect NSAID‐induced ulcer in Charles Foster rats by increasing the antioxidative factors, mucus and the total gastric tissue sulfhydryl group.^145^ The ethanol extract of betel leaves and the isolated compound allylpyrocatechol were found to have excellent healing properties against indomethacin‐induced stomach ulceration in rats (Sprague‐Dawley). The extract reduced the ulcer index by 93.4%, accelerated ulcer healing, improved the mucin content of gastric tissues, showed normal levels of malondialdehyde (MDA) and protein, and also increased levels of SOD and CAT levels.^146^, ^147^ The antiulcer activity of *P*.* betle* was also investigated in HCl‐ethanol, acute stress and pylorus ligation models in Wistar rats and Swiss albino mice using the hydroalcoholic extract of leaves. The results showed a decrease in ulcer index, gastric fluid volume, and an increase in gastric pH. An experiment was carried out using HAE (hot aqueous extract) and CEE (cold ethanolic extract) of betel leaves to assess gastroprotective efficacy in gastric ulcer induced by ethanol in crossbreed albino rats. When HAE and CEE were administered orally, they showed remarkable protection against gastric damage induced by absolute ethyl alcohol in a dose‐dependent manner by increasing the adhesion of the mucus content to the wall of the gastric mucosa and inhibiting the volume of gastric acid.^31^ All these experiments proved the traditional claim that *P*.* betle* could be an excellent gastroprotective and antiulcerogenic agent in therapeutics.

### 9.8 Antihyperglycaemic activity

The antihyperglycaemic efficacy of *P*.* betle* was investigated in streptozotocin‐diabetic albino Wistar rats. Leaf suspension was administered orally at a dose of 75 and 150 mg/kg body weight, for 30 days and the glucose level in plasma and glycosylated haemoglobin, liver hexokinase and gluconeogenic enzyme activities were tested. The result showed a decrease in blood glucose level from 205.00 mg/dl to 151.30 mg/dl, reduced glycosylated haemoglobin level, decreased liver fructose‐1,6‐bisphosphatase and glucose‐6‐phosphatase activity and also increased liver hexokinase in a dose‐dependent mode.^149^ The effect of betel leaf methanolic extract on *in vitro* protein glycation was investigated using a BSA (Bovine Seram Albumin)‐glucose model. The methanol extract inhibited glucose‐induced protein glycation in different stages and also inhibited the dose‐dependent modification of the thiol group and carbonyl formation. The early stage or the formation of amadori products was identified by haemoglobin‐δ‐gluconolactone, middle stage or the formation of the oxidative cleavage product was identified by BSA‐methylglyoxal, and the last stage or the production of advanced glycation ends (AGE) was identified by BSA‐glucose.^150^ Fatmawati and Shimizu, by *in vitro* aldose reductase assay, identified that the ethanolic extract of betel leaves can inhibit human recombinant aldose reductase (HRAR), which is the key compound in the polyol signalling pathway in converting glucose to sorbitol,therefore, long‐term diabetic complications develop contradictory with the value of IC_50_ 18.8 μg/ml.^136^

### 9.9 Antihyperlipidaemic activity

The Wistar rat brain when treated with ethanol showed that lipid peroxidation, lipids and turbulence in antioxidant protection are increased. Different doses of administration of the aqueous extract of the *P*.* betle* leaf showed improvement in toxicity symptoms. Co‐administration of the aqueous extract at the 300 mg/kg dose rate showed the highest activity and appreciably abridged the levels of lipids such as phospholipids, free fatty acids and cholesterol, and also reduced markers for lipid peroxidation such as TBARS and hydroperoxides, and increased antioxidants, such as SOD, reduced GSH, vitamin E, vitamin C, CAT and GSH peroxidase.^151^ The hypolipidaemic activity of betel leaf was evaluated in hyperlipidaemic rats fed a high‐fat diet. The leaf methanolic extract showed depletion in TC (total cholesterol), TG (triglycerides), LDL (low‐density lipoprotein) and VLDL (very low‐density lipoprotein‐cholesterol) activities in serum.^152^

### 9.10 Anti‐atherogenic activity

Atherosclerosis is a major health problem, which subsequently leads to cardiovascular disease caused by hypercholesterolemia. Venkadeswaran et al. studied the anti‐atherogenic potential of the *P*.* betle* plant and the active constituents present in it in the Triton WR‐1339‐induced hypercholesterolaemic Wistar rat. The betel leaf ethanol extract at a dose of 500 mg/kg w and its constituent eugenol at a dose of 5 mg/kg wt for 7 days of administration ameliorated hypercholesterolemia‐induced high levels of TC, TG, LDL and VLDL and low levels of enzymatic and non‐enzymatic antioxidants as standard lipid lowering drug, lovastatin.^153^ In another experiment, the authors fed Wistar rats an atherogenic diet and biochemical and histopathological experiments exhibited that the ethanolic leaf extract and the active constituent eugenol are capable of lowering the amount of TG, TC, LDL‐cholesterol and VLDL‐cholesterol in serum and liver tissue. The extract and eugenol also reduced AST, alkaline phosphatase, ALT, enzymes for lipid metabolization and lactate dehydrogenase in serum, decreased the antioxidant enzyme, and induces malondialdehyde in liver tissue and hemolysate.^154^

### 9.11 Cardioprotective activity

The effectiveness of *P*.* betle* in cardioprotection was evaluated in rats with isoproterenol (ISP)‐induced myocardial infarction. Oral administration of betel leaf hydroalcoholic extract significantly modulated the haemodynamic of systolic pressure, diastolic pressure, mean arterial pressure (DAP, MAP and SAP) and parameters for ventricular function such as contractility (+LVdP/dt) and relaxation (LVdP/dt), heart rate (HR); the extract restored the level of catalase (CAT), glutathione peroxidase (GPx), GSH and SOD, decreased leakage of creatine phosphokinase‐MB (CK‐MB) isoenzyme of and LDH, reduced lipid peroxidation in the heart showing a protection effect against ISP‐induced myocardial infarction.^155^ *Piper betle* was found to prevent oxidative cardiac cell injury through an *in vitro* study using the rat heart cell line H9c2 incubated with H_2_O_2_. The leaf extracted using ethyl acetate and the isolated bioactive component eugenol protected against oxidative stress induced by H_2_O_2_, decreased intracellular ROS and apoptosis and improved the cellular defence system at a dose of 10 μg/ml.^156^

### 9.12 Antifertility activity

The first report on the antifertility potential of *P*.* betle* was probably from Tewari et al., who found that the alcoholic extract of the betel stalk can reduce the number of pups; anti‐oestrogenic property recorded in adult male, female rats and rabbits. Gentle progestational action was also found in oestrogen‐primed immature rabbits with few types of follicle depressant in their regressive phase.^157^ To evaluate the antifertility efficacy of *P*.* betle*, alcoholic extract of leaf stalks is administered orally to male Swiss albino mice at a dose of 500 mg initially for 30 days and after that a dose of 1000 mg/kg body weight for another 30 days per animal per day. After 60 days of treatment, fertility was reduced to 0%. The extract suppressed sperm mobility and cauda epididymal sperm count, reduced fructose content in the seminal vesicles and weights of reproductive organs, and also increased cholesterol in the testes. The altered parameters were found to recover after discontinuation of the extract, suggesting *P*.* betle* as a contraceptive agent without altering hormonal balance.^158^ The antifertility efficacy of betel petiole extract was studied in female albino Wistar rats. Petiole ethyl alcohol extract at a dose of 100 mg/day/rat for 30 days showed a reduction in fertility, reproductive organ weights, oestrogen level, litter number, serum glucose concentration, acid phosphatase, SGOT and SGPT activity,increased cholesterol and ascorbic acid activity. The extract revealed that cholesterol was not used and there was no mobilization of ascorbic acid, irregular oestrus cycle and no change in haematological parameters.^159^ The application of methanol and the aqueous extract of betel leaf extract on the female Wistar rat revealed an irregular and prolonged oestrous cycle, which results in infertility.^68^ *In silico* study of the antifertility effect of *P*.* betle* root extract containing piperolactam A exhibited potential contraceptive activity with high binding affinity to the oestrogen and progesterone receptor (8.9 and 9.0 Kcal/mol, respectively), the binding site showed more hydrogen binding to the receptor than Rohitukine and OrgC.^160^ Shah and Jhade studied the antifertility effect of the betel plant on adult female Wistar rats using the water, petroleum ether and ethanol extract of the whole plant. The results showed significant antifertility potential with anti‐implantation and abortifacient activity, reduced level of follicle stimulating hormone (FSH), luteinizing hormone (LH), progesterone, anti‐oestrogenic activity and irregular oestrous cycle.^161^ ^,^ ^162^

## 10 ANTIMICROBIAL ACTIVITIES

The following section presents the antibacterial and antifungal properties of the plant (Table 4).

**TABLE 4:** Antimicrobial activities of *P*.* betle*

Effect	Extract (isolates)	Active against	Result	Reference
Antibacterial activity	stalk ethyl acetate ethanol, hexane, benzene extract	*Vibrio cholerae Ogawa, Staphylococcus aureus, Diplococcus pneumoniae*, and *Klebsiella aerogenes*	ethyl acetate, ethanol extract showed significant activity, hexane, and benzene extract showed moderate activity	^165^
	Methanolic and aqueous extract of leaves	*Pseudomonas aeruginosa, P. testosteroni, P. pseudoalcaligenes, Staphylococcus aureus, S. epidermidis, S. subflava, Proteus mirabilis, Pr. vulgaris, Pr. morganii, B. cereus, B. subtilis, B. megaterium, Citrobacter freundii, Micrococcus flavus, Alcaligenes fecalis, Enterobacter aerogenes, Salmonella typhimurium, K. pneumoniae, Escherichia. coli, Streptococcus fecalis, St. cremoris, St. agalactiae*	methanol extract is more potent than aqueous extract	^166^
	essential oil	*S. aureus*	potential antibacterial activity	^167^
	ethanol extract of leaves	*S*. *aureus*, *Pseudomonas aeruginosa*, and *K*. *pneumoniae*, *Pr*. *vulgaris*	potent antibacterial activity with MIC range of 25 µg to 40 µg	^170^
	essential oil from leaves of vellaikodi variety	*S. aureus, St. mutans, Lactobacillus acidophilus*	potential antibacterial activity	^169^
	ethanol and aqueous extract of leaves	*B. subtilis, S. aureus, Micrococcus luteus, E. coli, P. aeruginosa*	ethanol extract is more potent than aqueous extract. The water extract did not show activity against *E*. *coli* and *P*. *aeruginosa*	^171^
	water, methanol, ethyl acetate and petroleum ether extracts of leaves	*St*. *pyogenes*, *S*. *aureus*, *Pr*. *vulgaris* and *E*. *coli*	all extracts showed antibacterial activity against all tested bacteria	^32^
	cold aqueous, ethanol, methanol, and ethyl acetate extracts of leaves (Desawari, Desi, Bangladeshi and Jaleswar varieties)	*P*. *aeruginosa*, *S*. *aureus* and *E*. *coli*	all varieties in all solvents are effective against all bacteria; Bangladeshi and Jaleswar, the varieties in Ethanol, Ethyl Acetate, and Methanol solvents were most effective	^172^
	essential oil from leaves of two varieties Bangladeshi and Deshwari	*S. aureus, St. epidermidis, K. pneumoniae*	potential antibacterial activity	^168^
	crude aqueous extract diluted in ethanol	*E. coli, K. pneumoniae, Proteus* sp., *P. aeruginosa, Vibrio cholerae, S. aureus, St. faecalis*	most bacteria were found to be susceptible with the highest bactericidal activity towards *E*. *coli*, *P*. *aeruginosa* and *S*. *aureus*	^174^
	methanol extract of leaves (eugenol, hydroxychavicol)	*Bacillus*sp., *Enterococcus faecalis*, *S*. *aureus*, *St*. *agalactiae*, *Aeromonas hydrophila*, *E*. *coli*, *K*. *pneumoniae*, *P*. *aeruginosa*, *V*. *alginolyticus*	promising concentration dependant antibacterial activity	^175^
	ethanol extract from leaves	*S*. *aureus* from conjunctivitis patient	potential antibacterial activity	^254^
	essential oil from fresh and cured leaves	*Mycobacterium smegmatis, S. aureus* and *P. aeruginosa*	cured leaf essential oil exhibited higher antimicrobial activity towards *M*. *smegmatis*	^1^
	ethanol extract of leaves	*S. simulans, S. chromogenes, S. mitis, St. dysgalactiae, St. agalactiae, St. uberis, St. sanguinis*	antibacterial effect at MIC 12.5 mg/ml	^173^
	crude water extract of young and mature leaves	*St*. *agalactiae* and *E*. *coli*	both extracts showed antibacterial activity; 30% extract of young leaf showed the highest activity against *S*. *agalactiae*	^176^
	acetone and ethanol extract of leaves (Barguna and Moheshkhali) varieties	*B*. *cereus*, *S*. *aureus* and *E*. *coli*	Barguna showed MIC of 2.12 to 4.25 mg/ml and Moheshkhali showed MIC 2.12–8.5 mg/ml	^177^
Antifungal activity	methanolic extract	*Candida tropicalis*	potential antifungal activity	^166^
	essential oil, methanol and aqueous extract	*Candida albicans*and *Malassezia pachydermatis*	potential antifungal activity	^167^
	leaf aqueous extract, chloroform fraction (hydroxychavicol)	*C. albicans, C. glabrata, C. krusei, C. parapsilosis, C. tropicalis, C. neoformans, Aspergillus flavus, A. fumigatus, A. niger, A. parasiticus, E. floccosum, M. canis, M. gypsium, T. mentagrophytes, T. rubrum*	concentration‐dependent antifungal activity against all fungi and inhibited biofilm of *C*. *albicans*	^186^
	Leaf extract by hydrodistillation	*C. albicans* and *Saccharomyces cerevisiae*	potential antifungal activity	^169^
	essential oil from leaves	*C. albicans, C. rugosa, Saccharomyces cerevisiae, A. flavus*	potential antifungal activity	^168^
	plant extract and methanol fraction	*A. flavus, C. albicans, Microsporum canis, Trichophyton mentagrophytes* and *T. rubrum*	antifungal activity against all fungi with maximum activity against *A*. *flavus*	^187^
	leaves crude extract and chloroform fraction (hydroxychavicol)	*Colletotrichum gloeosporioides, Rhizoctonia solani, Fusarium oxysporum* f. sp. *cubense, Sphaceloma ampelinum, C. capsici*	concentration‐dependent fungicidal and fungistatic activity	^188^
	leaf essential oil	*C. albicans*	moderate antifungal activity with MIC 0.4%	^183^
	ethanol extract of leaves	*C. albicans*	good anticandidal activity with MIC values 125 μg/ml	^184^
	ethanol extract of leaves	*A. flavus*	complete inhibition of fungal mycelia	^185^
	leaf extract	*C. albicans*	good antifungal activity	^182^

### 10.1 Antibacterial activity

The global epidemic of infectious diseases caused by microbes has a high mortality rate, resulting in a high global health burden. Antimicrobial resistance and the lack of novel vaccines make infectious diseases one of the greatest threats to human health globally. Various factors are contributing to the rise in antibiotic resistance among human invasive organisms.^163^, ^164^ The antimicrobial efficiency of the *P*.* betle* leaf stalk was studied against the human pathogenic bacteria *Staphylococcus aureus*, *Vibrio cholerae Ogawa*, *Klebsiella aerogenes* and *Diplococcus pneumoniae*. Among the extracts, the ethyl acetate and ethanol extracts exhibited remarkable activity, the hexane and benzene extracts exhibited moderate activity towards the majority of the bacteria.^165^ Nair and Chanda tested the antibacterial effect of betel leaf against several gram +ve and gram‐ve bacteria *Pseudomonas aeruginosa*, *P*.* testosteroni*, *P*.* pseudoalcaligenes*, *Staphylococcus aureus*, *S*.* epidermidis*, *S*.* subflava*, *Proteus mirabilis*, *P*.* vulgaris*, *P*.* morganii*, *B*.* cereus*, *B*.* subtilis*, *B*.* megaterium*, *Citrobacter freundii*, *Micrococcus flavus*, *Alcaligenes faecalis*, *Enterobacter aerogenes*, *Salmonella typhimurium*, *Klebsiella pneumoniae*, *E*.* coli*, *Streptococcus faecalis*, *St*. *cremoris* and *St*. *agalactiae* and found that methanol extract is more potent than aqueous extract in comparison with the standard drug Piperacillin and gentamicin.^166^ Essential oil of betel leaves of the Vellaikodi, Bangladeshi and Deshwari varieties showed potential antibacterial activity against *S*.* aureus*, *St*.* mutans*, *Lactobacillus acidophilus*, *St*.* epidermidis*, *K*. *pneumoniae e*.^167^, ^168^, ^169^ Antibacterial activity was found with a MIC range of 25–40 µg against *S*.* aureus*, *Pseudomonas aeruginosa* and *K*.* pneumoniae*, *P*. *vulgaris* using leaf ethanol extract.^170^ Kaveti et al. evaluated the antibacterial efficacy of leaf ethanol and aqueous betel extracts against *S*.* aureus*, *Micrococcus luteus*, *B*.* subtilis*, *P*. *aeruginosa* and *E*. *coli* and found that ethanol leaf extract is more potent in efficacy than aqueous extract, while water extract showed no efficacy against *E*. *coli* and *P*. *aeruginosa*.^171^ The methanol, water, petroleum ether and ethyl acetate extracts of leaves found to restrict the growth of *St*.* pyogenes*, *S*.* aureus*, *P*. *vulgaris*, *E*.* coli*, *P*.* aeruginosa*, *Bacillus* sp., *Enterococcus faecalis*, *St*.* agalactiae*, *Aeromonas hydrophila*, *K*.* pneumoniae*, *Vibrio cholerae*, *V*.* alginolyticus*, *S*.* simulans*, *S*.* chromogenes*, *S*.* mitis*, *St*.* dysgalactiae*, *St*.* agalactiae*, *St*.* uberis*, *St*. *sanguinis*, *K*.* pneumoniae e*, *Proteus* sp., *S*. *aureus* and *St*. *faecalis* in different experiments.^32^, ^172^ ^,^ ^173^ ^,^ ^174^, ^175^. Lubis and Marlisa collected *S*. *aureus* from patients with swab of the conjunctivitis patients and observed that the ethanol extract of the leaves effectively inhibited bacteria.^254^ Essential oils from fresh and cured leaves were used to investigate the bactericidal activity of *P*.* betle*. The results showed that the cured leaf essential oil exhibited higher antimicrobial activity towards *M*. *smegmatis*.^1^ Surjowardojo et al. tested the crude water extract of young and mature leaves in *St*. *agalactiae* and *E*. *coli* and found that both extracts showed antibacterial activity, while 30% of the young leaf extract showed the highest activity against *S*. *agalactiae*.^176^ A recent experiment compared antibacterial efficacy between acetone extract and ethanol extract from leaves (Barguna and Moheshkhali) against the varieties of *S*.* aureus*, *E*. *coli* and *B*. *cereus*. The Barguna showed a MIC (minimum inhibition concentration) value of about 2.12 to 4.25 mg/ml, and the Moheshkhali variety showed a MIC of 2.12 to 8.5 mg/ml.^177^ The ethanol extract of betel leaves also showed antibacterial efficacy against foodborne bacteria such as *E*. *coli*, *Shigella dysenteriae*, *Staphylococcus aureus* and *Vibrio cholera* with MIC values in the range of 0.625–0.75% (w/v).^178^ The inhibition activity of food and waterborne pathogens from betel leaf was evaluated for multidrug resistant *Staphylococcus aureus*, *Salmonella typhi*, *P*. *aeruginosa*, *B*.* cereus*, *E*. *coli* and *B*. *subtilis*. Different solvent extracts, *viz*. methanol, ethanol and water showed significant antibacterial potency against all bacteria tested.^179^ The antibacterial experiment of *P*.* betle* showed that betel leaf extract in n‐hexane and ethyl acetate promisingly inhibits fish pathogens such as *Aeromonas hydrophila*, *Vibrio alginolyticus* and *Edwardsiella tarda*, demonstrating the application of betel extract in fish preservation.^180^, ^181^

### 10.2 Antifungal activity

Various preclinical studies proved the antifungal potential of *P*.* betle* against a number of fungi by different solvent extracts. Essential oils and ethanolic extract from leaves showed potential antifungal activity against *Candida albicans*.^182^, ^183^, ^184^ Complete inhibition of *Aspergillus flavus* fungal mycelia^185^ by ethanol extract and inhibition of *Candida tropicalis* ^166^ were also observed in two different experiments. Essential oil, methanolic and aqueous leaf extracts of betel against *Candida albicans* and *Malassezia pachydermatis* ^167^ and leaf extract by hydrodistillation against *Saccharomyces cerevisiae* and *Candida albicans* ^169^ exhibited significant antifungal activity. Ali et al., tested leaf aqueous extract and chloroform fraction (isolated compound hydroxychavicol) against *C*.* albicans*, *C*.* glabrata*, *C*.* krusei*, *C*.* parapsilosis*, *C*.* tropicalis*, *C*.* neoformans*, *A*.* flavus*, *A*.* fumigatus*, *A*.* niger*, *A*.* parasiticus*, *M*.* canis*, *M*.* gypsium*, *T*.* mentagrophytes*, *T*. *rubrum* and *E*. *floccosum*. The result showed concentration‐dependent antifungal activity against all fungi, and inhibition of the *C*. *albicans* biofilm was also observed.^186^ The potential antifungal efficacy of the essential oil and the methanol extract of betel leaves was found in *C*.* rugosa*, *C*.* albicans*, *A*.* flavus*, *Saccharomyces cerevisiae*, *Microsporum canis*, *Trichophyton mentagrophytes and T*. *rubrum*.^168^, ^187^ In another experiment, crude leaf extract and chloroform fraction containing hydroxychavicol applied on *Colletotrichum gloeosporioides*, *Rhizoctonia solani*, *Fusarium oxysporum* f. sp. *cubense*, *Sphaceloma ampelinum and C*. *capsici* and the result showed concentration‐dependent fungicidal and fungistatic activity.^188^ The ethanol extract of *P*.* betle*, when tested against foodborne fungi *Aspergillus niger*, *A*. *oryzae* and *Penicillium s* sp. in agar diffusion assay, exhibited complete fungal inhibition at a concentration of >1.50% (v/v).^189^ The addition of betel essential oil at a safe concentration to apple juice and tomato paste was also found to improve antioxidant capacity and inhibit microbial growth, such as *Aspergillus flavus* and *Penicillium expansum*, enhancing shelf life under refrigerator conditions.^190^, ^191^

### 10.3 Antiparasitic activities

The following section presents the antiparasitic (anthelmintic, anti‐protozoan and antifilarial) activities of *P*.* betle* (Table 5).

**TABLE 5:** Antiparasitic activities of *P*.* betle*

Anthelmintic property	leaf extract	*Pheretima posthuma*	required less time for paralysis and death compared albendazole	^193^
	stem extract	*Pheretima posthuma*	caused death	^192^
	leaf extract	*Eisenia fetida*	less time for paralysis and death	^194^
	essential oil from leaves	*Ascaridia galli*	significant anthelmintic activity	^195^
Anti‐protozoan activity	chloroform leaf extract	*Giardia intestinalis*	anti‐giardial activity with MIC 250 (μg/ml) and IC_50_ value 51.57 (μg/ml)	^196^
	ethanol extract of leaves	*Leishmania donovani*	inhibited promastigotes and amastigotes by apoptosis and morphological changes, mitochondrial membrane potential loss, DNA fragmentation, and cell‐cycle arrest at G0/G1 phase	^197^
	methanol extract of leaf extract (Bangla Mahoba variety)	*Leishmania donovani*	inhibited promastigotes and amastigotes, accelerated apoptosis, generated ROS targeting mitochondria	^200^
	leaves ethanol extract	*Leishmania donovani*	inhibited promastigotes at a concentration of 8.42 ± 2.03 mg/ml and 50.2 ± 13.75 mg/ml after 24 h and 48 h, respectively	^199^
	root extract	*Leishmania donovani*	Inhibited axenic and intracellular amastigotes	^198^
	methanol extract of leaves	*Plasmodium berghei*	significant (*p* < 0.05) schizonticidal activity at a dose of 50–400 mg/kg in ICR mice	^201^
	leaf extract	*Toxoplasma gondii*	25 µg/ml inhibited parasite invasion into host human foreskin fibroblast cells, reduced parasite burden in the brains of BALB/c mice	^202^
	leaf extract	*Neospora caninum*	inhibit parasite growth in human foreskin fibroblast cells, increased survival of C57BL/6 mice	^203^
Antifilarial activity	crude methanol extract, n‐hexane, and chloroform fractions	*Brugia malayi*	suppressed microfilaraemia, potential macrofilaricidal efficacy, sterilized female worms, increased antifilarial IgG antibody	^204^

#### 10.3.1 Anthelmintic property

Helminths produce substances, which have substantial toxicity towards humans, that are found in foods acquired from livestock, causing a serious hazard to human health, including lymphatic filariasis or elephantiasis, onchocerciasis, and schistosomiasis. The anthelmintic activity of *P*.* betle* was studied using aqueous and ethanol extracts of the stem against the adult Indian earthworm *Pheretima posthuma*. The results showed that the time required to cause paralysis and death is less in ethanol extract and aqueous extract than in the standard drug albendazole.^192^ Akter et al. also observed the same activity when using leaf methanol extract instead of stem extract.^193^ The anthelmintic efficacy of the crude aqueous leaf extract of *P*.* betle* was also evaluated in adult earthworm *Eisenia fetida*. The result expressed anthelmintic activity in terms of less time for paralysis and death of the earthworm.^194^ The anti‐helmintic activity of the essential oil of *P*.* betle* from leaves was also found to inhibit the burden of *Ascaridia galli* in poultry birds.^195^

#### 10.3.2 Anti‐protozoan activity

Giardiasis is the most common protozoan parasitic infection of the human intestine. Anti‐giardial activity was observed using chloroform extract of *P*.* betle* leaves against trophozoites of *Giardia intestinalis* with MIC 250 (μg/ml) and IC_50_ value 51.57 (μg/ml).^196^ Leishmaniasis is also a protozoan parasitic infection caused by *Leishmania* that results in a broad spectrum of clinical representation with significant morbidity and also mortality throughout the world. Ethanolic betel leaf extract showed antileishmanial potency towards both promastigotes with IC_50_ value of 9.8 and against amastigotes with IC_50_ value 5.45 μg/ml of *Leishmania donovani*, mediated by apoptosis and morphological changes, loss of mitochondrial membrane potential, DNA fragmentation and cell cycle arrest in the sub‐G_0_/G_1_ phase.^197^ Antileishmanial activity was also reported in which the use of leaf ethanol extract and root extract inhibited promastigotes and amastigotes of *Leishmania donovani*.^198^, ^199^ Inhibition of promastigotes and amastigotes, acceleration in apoptosis, and ROS generation targeting *Leishmania* mitochondria was also observed using methanol extract of betel leaves of Bangla Mahoba variety.^200^ *Plasmodium berghei*, the causal agent for human malaria, is parasitic protozoa of mosquito. The betel leaf extract showed notable schizonticidal activity (*p* < 0.05) and an antiplasmodial effect at a dose of 50–400 mg/kg in ICR mice.^201^ Leesombun et al. described that the betel leaf extract is capable of inhibiting the invasion of the *Toxoplasma gondii* parasite into human foreskin fibroblast cells at a dose of 25 µg/ml dose and reduced parasite burden in the brains of BALB/c mice.^202^ The authors also found that betel leaf extract can inhibit the growth of *Neospora caninum* parasites in human foreskin fibroblast cells and increase the survival of C57BL/6 mice.^203^

#### 10.3.3 Antifilarial activity

*In vivo*antifilarial activity of *P*.* betle* was evaluated using crude methanolic extract, chloroform, and n‐hexane extracts were administered at different doses to Balb/c mice. All extracts showed antigen‐specific immune response, increased antifilarial IgG antibody and also suppressed microfilaraemia, showed potential macrofilaricidal efficacy, and induced sterilization of female worms.^204^

### 10.4 Insecticidal activities

The insecticidal activity of *P*.* betle* was evaluated using an aged grain assay against bean weevil (*Sitophilus zeamai*s), lesser grain borer (*Rhyzopertha dominica*) and cowpea weevil cowpea weevil (*Callosobruchus maculatus*). The 30% volatile oil dust formulation exhibited toxicity against adult insects, prevented the survival of adult *C*. *maculatus*, and up to 52% protected corn against *S*. *zeamais* and *R*. *dominica*; also inhibited living and emerging progeny.^205^ Nair and Kavrekar found that the methanol extract of betel leaves can exhibit good insecticidal activity against insects such as *Bruchus pisorum*, *Tribolium castaneum* and *Sitophilus oryzae*.^206^ The following section presents the insecticidal activities of *P*.* betle* (Table 6).

**TABLE 6:** Insecticidal, larvicidal and adulticidal activities of *P*.* betle*

Insecticidal activity	essential oil	*Sitophilus zeamais motschulsky, Rhizopertha dominica, Callosobruchus maculatus*	inhibition of living and emerging progenies	^205^
	methanol extract of leaves	*Bruchus pisorum, Tribolium castaneum, Sitophilus oryzae*	good insecticidal activity	^206^
Larvicidal activity	essential oil and methanol extract of leaves	*Aedes aegypti*	for essential oil at 2 h and 24 h LD_50_ value of 86 and 48 ppm; for methanol extract at 2 h and 24 h LD_50_ value of 153 and 125 ppm, respectively	^167^
	methanol extracts of leaves	*Aedes aegypti*	LC_50_ values 313.58 and 122.99 ppm, respectively after 24 and 48 h	^207^
	essential oil	*Aedes aegypti*	24 h exposed, LC_50_ = 13, l ppm – For the 48‐h exposed, LC_50_: 1l,2 ppm	^208^
	essential oil	*Aedes aegypti*	for larvicide activity, the LC_50_ values at 1 h, 24 h and 48 h are 183, 92.7 and 59.8 ppm	^210^
	essential oil from betle leaf	*Chrysomya bezziana*	4% essential oil killed all first instar larvae in 2 h while killing second instar larvae in 4 h	^209^
	essential oil from betle leaf	*Chrysomya megacephala*	3 and 4% essential oil killed 100% larvae in 3.5 h	^211^
	methanol extract of leaves	*Drosophila melanogaster*	dose‐dependent larvicidal activity with a reducing effect on the nucleic acid and protein content	^206^
Mosquito adulticidal activity	essential oil	*Aedes aegypti*	concentration of 2.5 μl/ml, caused 100% mortality in adult mosquitoes within 15–30 min	^210^

### 10.5 Larvicidal property

The mosquito larvicidal activity of *P*.* betle* against *Aedes aegypti was evaluated* using methanol extract and essential oil of the leaves. For essential oil, the LD_50_ values were found to be 86 and 48 ppm at 2 and 24 h; for the methanol extract, the LD_50_ values at 2 and 24 h are 153 and 125 ppm, respectively.^167^ Tennyson et al. observed larvicidal activity with LC_50_ values 313.58 and 122.99 ppm, respectively, after 24 and 48 h using leaves methanol extract.^207^ The essential oil of the betel leaves can also inhibit the larval growth of *Aedes aegypti*. When the third instar larvae were exposed for 24 h, the LC_50_ value found l3, l ppm, and for the 48‐h exposure, the LC_50_ value is 1l, 2 ppm.^208^ Essential oil from obtained betel leaf was also treated on *Chrysomya bezziana* larvae, and the result showed that 4% essential oil killed all first instar larvae in 2 h while killing the second instar larvae in 4 h.^209^ Larvicidal activity using *P*.* betle* essential oil was also observed with LC_50_ 183 ppm 92.7 ppm and 59.8 ppm and LC_90_ 637 ppm 525 ppm and 434.7 ppm, after 1, 24 and 48 h after treatment, respectively.^210^ Another experiment showed that 3 and 4% essential oils are also capable of killing 100% *Chrysomya megacephala* larvae in 3.5 h.^211^ *Drosophila melanogaster* larvae were also found to be killed with the administration of methanol extract of the leaves in a dose‐dependent manner by reducing the effect on the nucleic acid and protein content.^206^

## 11 MISCELLANEOUS ACTIVITIES

### 11.1 Antiplatelet activity

Three compounds (B‐sitosterol, ursonic acid and 3B‐acetyl ursolic acid) from betel root extract were isolated and identified to evaluate the antiplatelet activity of *P*.* betle* arachidonic acid (AA), platelet activation factor (PAF) and Adenosine diphosphate (ADP) induced human platelet aggregation (PA). An *in vitro* study showed that all three compounds have potency in inhibiting PA. The order of inhibition of AA‐induced PA inhibition is B‐sitosterol < 3B‐acetyl ursolic acid < ursonic acid. Only B‐sitosterol and ursonic acid have inhibitory activity towards PAF and inducing activity to PA, whereas B‐sitosterol only showed inhibitory activity against ADP‐induced PA.^212^ The antiplatelet activity of the aqueous extract of the inflorescence of *P*.* betle* was investigated in collagen‐induced and AA‐induced rabbit PA. *In vitro* treatment of the extract inhibited platelet aggregation induced by collagen and AA‐ with IC_50_ values of 207 and 335 µg/ml, respectively, inhibited the production of AA, collagen and thrombin‐induced thromboxane B2 (TXB2), induced by >90%, indicating that the extract of *P*.* betle* contains compounds that can inhibit platelet aggregation by ROS elimination or inhibition of TXB2 production (Table 3).^213^

### 11.2 Anti‐halitosis activity

Halitosis is the degradation of proteins and amino acids present in saliva, gingival cervical fluid or food retained in the teeth that causes bad breath or oral malodour due to microbial activity. The methanol extract and fractions of leaves (isolated compound allylpyrocatechol—APC) showed antibacterial activity against oral bacteria and reduced the production of volatile sulphur compound (VSC) by oral anaerobic bacteria using an *in vitro* saliva chip model. APC also potentially reduced methyl mercaptan and hydrogen sulphide and prevented periodontal infection.^214^

### 11.3 Antiallergic activity

To know about the antiallergic activity of the ethanol extract of *P*.* betle* leaf on the synthesis of GM‐CSF (granulocyte macrophage colony‐stimulating factor) and histamine by BMMC (murine bone marrow mast cells) and also the activity on the human lung epithelial cell line, BEAS‐2B mediated the secretion of eotaxin and IL‐8 was evaluated *in vitro*. Treatment with extract markedly reduced histamine and IgE‐mediated hypersensitive reaction‐mediated GM‐CSF production; it also inhibited eotaxin and IL‐8 secretion produced by an allergic reaction induced by TNF‐α and IL‐4. This experiment suggests that *P*.* betle* controls allergic diseases by inhibiting the production of allergic mediators and can be used as a therapeutic antiallergic agent.^215^

### 11.4 Anti‐asthmatic activity

The anti‐asthmatic activity of *P*.* betle* against 0.2% histamine‐induced bronchospasm in guinea pigs was evaluated using ethanol extract. Treatment of the extract, with a dose of 100 and 200 mg/kg bw, exhibited a prominent anti‐asthmatic effect with a prolonged latent period of convulsions compared to the standard antihistaminic drug, chlorpheniramine.^216^

### 11.5 Dermatological activities

To evaluate the dermatological activity of *P*.* betle*, *the* crude ethanolic extract of the leaves and the formulated cream were tested for certain zoonotic dermatophytic fungi, that is *Trichophyton mentagrophyte*, *Microsporum gypseum*, *Microsporum canis* and *Candida albicans*. The broth dilution and disc diffusion assay revealed significant antifungal activity with a range of IC_50_ values 110.44–119.00 µg/ml. The result suggests that *P*.* betle* has notable therapeutic importance for the treatment of dermatophytosis comparable to the ketoconazole drug, proving the traditional claim.^217^

### 11.6 Antihemolytic activity

The antihemolytic efficacy of the betel plant was investigated in an *in vitro* H_2_O_2_‐treated human erythrocyte model. Different solvent extracts, such as water, ethyl acetate, petroleum ether and methanol extracts from leaves, were used in the study, and the result showed reduced haemolysis without any toxicity as compared to ascorbic acid, taken as a positive control. Further lipid peroxidation was tested in terms of malonaldehyde production, showing reduced peroxidation in H_2_O_2_‐induced RBC (Red blood cell) cells by the effect of leaf extracts.^32^

### 11.7 Role in thyroid function

Panda and Kar in an experiment found that *P*.* betle* leaf extract showed a dual role on thyroid function in rats. The leaf aqueous extract was administered to Swiss albino male mice and changes in the concentrations of thyroid hormone, LPO (lipid peroxidation), SOD and CAT activity were investigated. Higher doses increased LPO concentration and decreased SOD and CAT activities. Higher doses decreased triiodothyronine (T_3_) and increased thyroxine (T_4_) concentrations, while the lowest dose increased T_3_ and decreased T_4_ concentrations.^218^

### 11.8 Immunomodulatory effect

Many *in vivo* and *in vivo* experiments were performed to prove *P*.* betle* as a novel immunomodulatory plant. The methanol extract of betel leaves in an *in vitro* study showed that the proliferation of peripheral blood lymphocytes was significantly induced by the suppression of phytohaemagglutinin in a dose‐dependent manner. Furthermore, the activity of *P*.* betle* was studied in mice that were immunized with sheep red blood cells using the extract at different dose levels to observe the cellular and humoral immune responses. The extract showed dose‐dependent suppression of T‐cell, B‐cell and immune response mediated by antibody, decrease in antibody titre, increase in inflammation suppression; delayed T‐cell‐mediated hypersensitivity reaction. The methanol extract prepared from betel leaves at a dose of 500 mg/kg showed immunosuppression that was in correlation with cyclophosphamide, an immunosuppressive drug (2 mg/kg) suggesting *P*.* betle* as a potent therapeutic agent for the treatment of various autoimmune disorders and immune disorders.^71^ The crude n‐hexane and methanol extracts of *P*.* betle* showed immunomodulatory effectiveness in Balb/c mice infected with *Brugia malayi*, a parasite of human lymphatic filaria. *In vivo* experiment showed enhancement in both humoral immune responses by increasing plaque‐forming cells and hemagglutination titre,enhanced cell‐mediated immune responses such as lymphoproliferation, delayed type of immune responses for hypersensitivity, macrophage activation; increased population of B cells (CD19 +) and T cells (CD4 +, CD8 +) and produced type‐1 and type‐2 cytokine responses.^204^

### 11.9 Radioprotective activity

Mitochondria from rat liver and plasmid DNA (pBR322), the two models were used to evaluate the radioactive property of the *P*.* betle* plant. Treatment of ethanol extract of leaves on *in vitro irradiated* mitochondria of rat liver and plasmid DNA (pBR322) prevented ray‐induced lipid peroxidation (thiobarbituric acid—TBA, reactive substrates of TBA, conjugated diene and LOOH) and DNA strand breaks; the extract also improved HO and SOD radical scavenging activity together with the lymphoproliferative property in a concentration‐dependent method.^55^

### 11.10 Anti‐acne activity

Acne, an inflammatory skin disease, caused by *Propionibacterium acnes* and *Staphylococcus aureus* due to blocking of polysebase. To evaluate the efficacy of *P*.* betle* against acne, a cream dose of betel leaf ethanol extract was prepared and applied to *P*. *acnes* and *S*. *aureus* using a disc diffusion process and the MIC was calculated. The result showed antibacterial efficacy with MIC values of 4.5% and 4.0%.^219^ Meinisasti et al. also showed that the cream formulation prepared from ethanol extract is effective against *P*. *acnes*.^220^ In another experiment, noisome gel containing essential oil from betel leaves was prepared which also inhibited *P*. *acnes* in Franz diffusion cell.^221^

## 12 TOXICITY PROFILE

An acute and chronic preclinical toxicity study was performed using the alcoholic extract of *P*.* betle* leaf stalk in different doses in mice and rats. Hematological, biochemical and chemical evaluations indicated that the alcoholic extract is devoid of any toxicity at the dose level of 100, 200 and 300 mg/kg bodyweight for 60 days and also interestingly 3200 mg/kg bw did not show any toxicity.^222^ An acute toxicity study in guinea pigs was studied by administering betel leaf extract that did not show death within 24 h of a dose of 100 and 200 mg/kg but at a dose, more than 300 mg/kg was found to exhibit 50% mortality, suggesting that doses of 100–200 mg/kg are safe.^223^ Venkateswarlu and Devanna reported that the leaf aqueous extract of *P*.* betle* up to the dose of 1000 mg/kg (po) body weight is safe when administered to Albino rats.^115^ Doses of up to 2000 mg/ kg were also found to be without toxicity in mice administered with hydroalcoholic betel leaf extract. No occurrence of death, no abnormal general symptoms, no effect of necropsy and histopathological lesions observed for 14 days after the methanol extract of betel leaf administered to ICR mice at a dose of up to 5000 mg/kg.^201^ De et al. also found that the ethanol extract of betel leaves is safe up to 2000 mg/kg bw without any toxicity or morbidity during the 14‐day observation period in Sprague‐Dawley rats.^143^ All of these studies suggest that *P*.* betle* is safe at higher doses and can be used as a therapeutic agent to treat various maladies.

## 13 NANOFORMULATIONS

Nanotechnology aims to synthesize materials with unique properties such as at least in one‐dimension, small size, surface charge, high surface energy, porosity and a large surface area/volume ratio, proving advantageous for catalysis and interacting with other molecules. Scientists have developed green chemistry methods with the synthesis of nanomaterials using different biological sources which are more sustainable, cleaner and eco‐friendly, non‐toxic, energy‐efficient, that eliminates the need for high energy, pressure, temperature, and needs no stabilizing, reducing and capping agents from outside.^50^, ^51^, ^224^. The petiole extract of P.* betle* leaf was used to synthesize stable silver nanoparticles with or without CTAB (cetyltrimethylammonium bromide) and SDS (sodium dodecyl sulphate). The polyphenolic groups contained in the leaf extract ^225^are the main agents responsible for the reduction of n Ag+ions into metallic Ag^0^ and also for stabilizing and capping. The morphology and crystalline phase were characterized by selected area electron diffraction (SAED) and transmission electron microscopy (TEM).^226^, ^227^. Green synthesis of silver nanoparticles from betel leaf extract was also confirmed and characterized by energy‐dispersive X‐ray analysis (EDX), X‐ray diffraction (XRD), scanning electron micrograph (SEM) and Fourier transform infrared (FTIR) studies.^228^ The ethanolic leaf extract of *P*.* betle* was also used for the successful synthesis of AuNPs (gold nanoparticles), which were characterized by TEM, Fourier transform infrared (FT‐IR), EDX and XRD. These nanoparticles were tested non‐toxic to MCF‐7 and HeLa (cancer) cell lines.^229^ Gadolinium‐doped titanium dioxide nanoparticles (GdT NPs) were also synthesized from the leaf of *P*.* betle* using the hydrothermal method. GdT NP showed high antibacterial activity against *S*. *aureus*, *E*. *coli* and *C*. *albicans* at 25µg/ml and also showed promising antioxidant activity in the DPPH radical scavenging method.^230^ The extract of the leaf of *P*.* betle* was also used in the synthesis of titanium dioxide nanoparticles (TiO_2_NP). TiO_2_ nanoparticles were characterized by TEM, XRD and FTIR, and antioxidant activity was evaluated using the DPPH assay, which showed promising antioxidant activity with the lowest IC_50_ value.^231^ The leaf of *P*.* betle* helps stabilize and capping in the phytofabrication of zinc oxide nanoparticles (PZnO). These PZnO exhibited antibacterial activity towards pathogens related to dental infections such as *Lactobacillus acidophilus* and *St*. *mutans* in the well diffusion test at low concentrations of 3.25 μg/ml and also demonstrated a high antioxidant efficacy of approximately 70% at a concentration of 200 μg/ml concentration in the DPPH assay.^232^ An experiment showed that *Piper betle* leaf extract‐mediated silver protein (core‐shell) nanoparticles (Ag NP) showed less toxicity against *Daphnia magna* than chemically synthesized AgNPs.^233^ Copper oxide nanoparticles (CuONPs) synthesized using *P*.* betle* leaf extract efficiently inhibited the growth of phytopathogens such as *Xanthomonas axonopodis* and *Ralstonia solanacearum* and also exhibited a cytotoxic effect on rat splenocytes by decreasing cell viability to 94% at 300 μg/ml.^234^ Silver nanoparticles coated with polyaniline (AgNP) synthesis from *P*.* betle* leaf extracts were evaluated for antimicrobial potency. The result exhibited 32.78 ± 0.64 mm inhibition zone for *S*. *aureus*, *a* maximum 29.55 ± 0.45 mm inhibition zone against *S*. *typhi*, 21.95 ± 0.45 mm for *P*. *aeruginosa* and 27.12 ± 0.38 mm for *E*. *coli* compared to the standard drug norfloxacin.^235^ Silver nanobioconjugates synthesized from the leaf extract of betel and its chief compound eugenol showed potent anticancer activity in lung adenocarcinoma (A549 cell line) with low viability and nuclear fragmentation in the MTT ‐ (3‐[4,5‐dimethylthiazol‐2‐yl]‐2,5 diphenyl tetrazolium bromide) assay and techniques for staining with orange acridine or ethidium bromide, also showed no toxicity against non‐cancerous human peripheral blood lymphocytes.^236^ Another experiment showed that silver nanoparticles synthesized using *P*.* betle* showed antifungal potency against *Fusarium solani* and *Alternaria brassicae* in a dose‐dependent method.^237^, ^238^ The Betel leaf extract was used to synthesize a silver‐gold nanocomposite (Ag‐Au NCPs) through the reduction of silver nitrate and gold chloride by the biological reduction method and was confirmed by XTD, SEM, FTIR and EDX. This bimetallic composite nanoformulation significantly inhibited *B*. *subtilis* and *K*. *Planticola* with higher antibacterial activity against *B*. *subtilis*.^228^ Green synthesized CaO calcium oxide nanoparticles from betel leaf extract, which are also able to inhibit *E*. *coli*, *P*. *aeruginosa*, *S*. *aureus* and *St*. *mutans* with the highest activity against *E*. *coli* and *St*. *mutans*. CaO nanoparticles also exhibited anticancer efficacy against the A549 cell line using an MTT assay with an IC_50_ value of 92.08 mg/ml.

## 14 CONCLUSIONS AND FUTURE PROSPECTS

*Piper betle* is a world‐known herbal cash crop of tremendous, social, economic and therapeutic importance and known as ‘green gold’. The mention of betel leaf is found in various ancient medicinal literatures, and the plant is still used in traditional and folklore medicinal systems. Traditional knowledge and preclinical studies revealed the use of *P*.* betle* which has potential multitherapeutic efficacy in various diseases such as cancer, inflammation, neurodegenerative disorders, asthma, dental and oral infections, allergy, thyroid, diabetes and skin diseases. Essential oils and extracts showed great results in antifertility, cardioprotection, hepatoprotection and antiplatelet. Various researchers have reported remarkable inhibition efficacy against insects, larvae, and improvement in microbial infections, parasitic infections. The plant contains a treasure of bioactive phytochemicals belonging to different classes such as phenol, tannin, terpenoid, alkaloids and flavonoids, which are responsible for healing of various diseases. The beautiful and pungent aroma of the plant is due to its phenolic and terpenoid compounds, which made betel an eminent flavouring agent. In addition to that, betel has a notably nutritional value and is considered GRAS (generally recognized as safe) for consumption.

*Piper betle* is found in many varieties and cultivars, and there is a problem in synonym and proper authentication; therefore, proper taxonomic identification of landraces is the most important criterion in research. Genetic and molecular markers must be used to differentiate the different varieties of betel. Different landraces contain different amounts and combinations of chemical constituents; therefore, efforts must be made to identify phytochemicals using modern extraction and detection techniques. Standardization and validation of chemical constituents for quantitative and qualitative evaluations must also be taken care of. As the quality and quantity of the chemical constituents vary with soil and environmental factors, therefore, the optimization of the highest‐yielding soil quality and factor must be studied for large‐scale commercial cultivation purposes. Although there are many reports available on preclinical treatment using *P*.* betle* in various diseases, the mechanism of the reaction is not mentioned in most reports. A computer‐aided drug discovery program can be used since it clarifies the molecular mechanisms, correlates the pharmacological responses with experimental data, and has a crucial role in boosting medical and pharmaceutical innovation. The docking analysis performed in this research provided valuable insights into the bindings of bioactive isolates towards many protein targets, including those involved in antidepressant, anti‐inflammatory and thrombolytic cascades.^237^ ^,^ ^238^ Broad‐spectrum clinical studies are also a major lacuna, which is incomplete in the pharmacological study. Therefore, more attention must be paid to the mechanism of action in different disease management in clinical studies that may open up an innovative avenue in therapeutics. Unexplored landraces must be further studied, and new varieties with a high number of bioactive compounds can be developed by the use of biotechnological methods. Furthermore, proper attention must be paid to the management of pests and diseases of betel plants and long‐term storage of leaves and extracts for commercial use.

The present review encompasses the traditional uses, preclinical and clinical aspects of *P*.* betle* with notes on its toxicological attributes and safety considerations. However, further studies are needed to explore its efficacy via proper elucidation of its underlying molecular mechanisms of action against various disease pathology, structure–activity relationships, bioavailability and synergism. In addition, well‐designed clinical studies involving statistically significant number of human patients are needed to assess the clinical significance of the plant preparations and the derived compounds. Lastly, its high abundance, low‐cost production, exportation and potential therapeutic aspects made the betel plant very distinguished throughout the world and opened myriad possibilities for future studies.

## CONFLICT OF INTEREST

The authors declare that there are no conflicts of interest.

## AUTHOR CONTRIBUTIONS

**Protha Biswas:**Conceptualization (supporting); methodology (supporting);writing ‐ original draft (supporting). **Uttpal Anand:** Conceptualization (lead); investigation (supporting); methodology (supporting);writing ‐ original draft (supporting). **Suchismita Chatterjee Saha:** Formal analysis (supporting); investigation (supporting); methodology (supporting);writing ‐ original draft (supporting). **Nishi Kant:** Methodology (equal). **Tulika Mishra:** Methodology (equal); writing, review, editing (equal). **Harison Masih:** Methodology (equal); writing, review, editing (equal). **Ananya Bar:** Methodology (equal); writing, review, editing (equal). **Devendra Kumar Pandey:** Data curation (supporting); formal analysis (supporting); writing, review, editing (supporting). **Niraj Kumar Jha:** Formal analysis (supporting); writing, review, editing (supporting). **Madhumita Majumder:** Formal analysis (supporting); writing, review, editing (supporting). **Neela Das:** Formal analysis (supporting); writing, review, editing (supporting). **Vijaykumar Shivaji Gadekar:** Methodology (equal); writing, review, editing (equal). **Mahipal S. Shekhawat:** Formal analysis (supporting); writing, review, editing (supporting). **Manoj Kumar:** Formal analysis (supporting); writing, review, editing (supporting). **Radha:** Data curation (supporting); writing, review, editing (supporting). **Jarosław Proćków:** Data curation (supporting); formal analysis (supporting); funding acquisition (lead); investigation (supporting); project administration (lead); resources (lead); supervision (lead); validation (supporting); visualization (supporting); writing, review, editing (supporting). **José M. Pérez de la Lastra:** Conceptualization (supporting); funding acquisition (lead); project administration (lead); resources (supporting); supervision (supporting); writing, review, editing (supporting). **Abhijit Dey:** Conceptualization (lead); formal analysis (lead); project administration (lead); resources (lead); supervision (lead); validation (supporting); visualization (supporting); writing, review, editing (supporting).

## Data Availability

Data sharing is not applicable to this article as no datasets were generated or analysed during the current study.
